# Adar3 Is Involved in Learning and Memory in Mice

**DOI:** 10.3389/fnins.2018.00243

**Published:** 2018-04-13

**Authors:** Dessislava Mladenova, Guy Barry, Lyndsey M. Konen, Sandy S. Pineda, Boris Guennewig, Lotta Avesson, Raphael Zinn, Nicole Schonrock, Maina Bitar, Nicky Jonkhout, Lauren Crumlish, Dominik C. Kaczorowski, Andrew Gong, Mark Pinese, Gloria R. Franco, Carl R. Walkley, Bryce Vissel, John S. Mattick

**Affiliations:** ^1^Garvan Institute of Medical Research, Sydney, NSW, Australia; ^2^St. Vincent's Clinical School, University of New South Wales, Sydney, NSW, Australia; ^3^Centre for Neuroscience and Regenerative Medicine, Faculty of Science, University of Technology Sydney, Sydney, NSW, Australia; ^4^St. Vincent's Centre for Applied Medical Research (AMR), Sydney, NSW, Australia; ^5^Institute for Molecular Bioscience, The University of Queensland, St. Lucia, QLD, Australia; ^6^Departamento de Bioquímica e Imunologia, Instituto de Ciências Biológicas, Universidade Federal de Minas Gerais, Belo Horizonte, Brazil; ^7^St. Vincent's Institute of Medical Research, Department of Medicine, St. Vincent's Hospital, The University of Melbourne, Fitzroy, VIC, Australia

**Keywords:** ADAR3, *Adar3*^*exon*3^ mouse model, RNA editing, learning and memory, Adarb2

## Abstract

The amount of regulatory RNA encoded in the genome and the extent of RNA editing by the post-transcriptional deamination of adenosine to inosine (A-I) have increased with developmental complexity and may be an important factor in the cognitive evolution of animals. The newest member of the A-I editing family of ADAR proteins, the vertebrate-specific ADAR3, is highly expressed in the brain, but its functional significance is unknown. *In vitro* studies have suggested that ADAR3 acts as a negative regulator of A-I RNA editing but the scope and underlying mechanisms are also unknown. Meta-analysis of published data indicates that mouse Adar3 expression is highest in the hippocampus, thalamus, amygdala, and olfactory region. Consistent with this, we show that mice lacking exon 3 of *Adar3* (which encodes two double stranded RNA binding domains) have increased levels of anxiety and deficits in hippocampus-dependent short- and long-term memory formation. RNA sequencing revealed a dysregulation of genes involved in synaptic function in the hippocampi of *Adar3*-deficient mice. We also show that ADAR3 transiently translocates from the cytoplasm to the nucleus upon KCl-mediated activation in SH-SY5Y cells. These results indicate that ADAR3 contributes to cognitive processes in mammals.

## Introduction

The human brain has evolved to enable unique cognitive capabilities and has tripled in size since the split from the chimpanzee lineage around 5 million years ago. Current hypotheses suggest that the advancement of human cognition most likely arose through the combined effects of the expansion of brain size and complexity, protein evolution (including new splice variants) and the emergence of RNA-based regulatory mechanisms that facilitate the epigenetic reformation of neural circuitry in response to experience (Barry and Mattick, 2012).

One of the RNA-diversification/regulatory mechanisms is RNA editing. Two main types of RNA editing are known, one involving the deamination of cytidine to create uridine (C-to-U), and the other, the deamination of adenosine to inosine (A-to-I). RNA editing not only alters the nucleotide sequence of target RNAs, but presumably also their structure-function relationships and interactions (Bass, 2002; Jantsch and Öhman, 2008).

The existence of RNA editing in mammals was first discovered in mRNAs for important neuroreceptors, such as the glutamate and serotonin receptors, and was initially thought to be an interesting but idiosyncratic mechanism to change their amino acid sequence (Maas et al., 2003), presumably to alter the electrophysiological properties of synapses in response to activity or other cues. Subsequently, transcriptome-wide analyses revealed that A-I editing is widespread in humans, occurring in thousands of transcripts, mostly in *Alu* sequences within intronic and intergenic sequences, varying in different tissues (Athanasiadis et al., 2004; Blow et al., 2004; Kim et al., 2004; Levanon et al., 2004; Ramaswami et al., 2012; Huntley et al., 2016). Although operating via a conserved mechanism (Jin et al., 2009), the rates of A-to-I editing have increased dramatically throughout vertebrate, mammalian and especially primate evolution, with RNA editing in humans being more than an order of magnitude higher than in mouse (Kim et al., 2004; Levanon et al., 2004). Moreover, more editing occurs in the human brain than in other primates (Paz-Yaacov et al., 2010).

Adenosine Deaminase Acting on RNA (ADAR) proteins, are responsible for the execution of the A-to-I RNA editing through hydrolytic deamination (Bass, 2002). Three ADAR enzymes (ADAR1-3) are encoded in the vertebrate genome, with ADAR3 being vertebrate-specific (Chen et al., 2000). Common to all ADARs is a C-terminal catalytic domain and multiple double-stranded RNA binding domains (Nishikura, 2010). In particular, ADAR3 shares 50% amino-acid sequence identity with ADAR2 (Melcher et al., 1996a) and is almost exclusively expressed in the nervous system, but its role is unknown. Unlike the other ADAR proteins, ADAR3 contains a novel arginine rich motif (herein, R-domain), which allows the binding of single stranded RNA (Chen et al., 2000), activity that may result in novel functions. Based on *in vitro* evidence, ADAR3 is suggested to act as a dominant-negative regulator of A-to-I RNA editing (Chen et al., 2000). The R-domain has also been proposed to serve as a functional nuclear localization signal (NLS) as it mediates interactions between ADAR3 and the Importin α protein complex enabling ADAR3 to locate to the nucleus (Maas and Gommans, 2009) where A-to-I RNA editing is believed to occur (Jin et al., 2009).

Due to the novelty and relatively unknown function of ADAR3 and its high expression in the nervous system, we investigated the role of this protein in cognition and behavior in ADAR3 deficient mice. We also demonstrate that mice lacking exon 3 of *Adarb2* (referred to herein as *Adar3*^*exon*3^), containing the double stranded RNA binding domains, display increased anxiety levels and impaired short and long-term hippocampus-dependent memory formation. RNA sequencing of mouse hippocampal tissue indicates roles for *Adar3* in synaptic function. Finally, our results also show that ADAR3 transiently translocates to the nucleus in response to neuronal activation in SH-SY5Y cells. Collectively, our data strongly suggest that ADAR3 is essential for correct cognitive functioning of the mammalian brain.

## Materials and methods

### Mouse lines: generation of *Adar3*^*exon*3^ mice

ES cells containing the targeted *Adarb2*^*tm*1*a*(*KOMP*)*Mbp*^ allele (IKMC project number 39714; hereafter referred to as *Adar3*^*tm*1*a*^) were generated by the trans-NIH Knock-Out Mouse Project (KOMP) and obtained from the KOMP Repository (www.komp.org). The *Adarb2*^*tm*1*a*(*KOMP*)*Mbp*^ allele contains a splice acceptor-beta-geo-polyA (SA-βgeo-pA) flanked by FRT sites located in intron 2 and loxP elements flanking exon 3 (KOMP designation: KO first allele (reporter-tagged insertion with conditional potential). Correct targeting was confirmed and transgenic mice were generated from embryonic stem cell clones DEPD0006_14_A03 & DEPD0006_14_A05. Animals were generated by the Australian Phenomics Network ES to Mouse service at Monash University. Positive mice were backcrossed to C57BL/6N background and genotyped by PCR. Primers P1 (located at the 3′ end of the 5′ homology arm) and P2 (located at the 5′ end of exon ENSMUSE00000465454) will amplify a product of 554 bp from the wild type allele. Primers P1 and P3 (located in the en-2 intron) will amplify a product of 246 bp from the targeted allele. P1 = 5′ CAATATACCACAACGAACATCTTTG 3′; P2 = 5′ GTCCCCAGGTTGCTCACATTTCG 3′; P3 = 5′ CAACGGGTTCTTCTGTTAGTCC 3′. PCR conditions were: an initial denaturation step at 94°C 3 min, followed by 35 cycles of 94°C 30 s, 57°C 30 s, 72°C 45 s and a final cycle of 72°C for 5 min.

Heterozygous *Adar3*^*tm*1*a*^ animals (identified in-house as ADAR2BlacZ line) were crossed with heterozygotes of a ubiquitous expressing Cre-recombinase mouse line (C57BL/6NTac-Gt(ROSA)26Sortm16(cre)Arte; Taconic). The Cre-recombinase gene was identified using the following primers, which yielded a 408 bp fragment: forward 5′ GCATTACCGGTCGATGCAACGAGTGATGAG 3′; and reverse 5′ GAGTGAACGAACCTGGTCGAAATCAGTGCG 3′. Thus, in addition to expressing Cre-recombinase, double heterozygous mice from the resulting cross, possessed one WT allele and one allele in which Cre-mediated deletion of the loxP-flanked portion of the *Adar3* gene had occurred. To maintain the strain background and breed out the Cre-recombinase allele, double heterozygous animals were then crossed back to WT mice from the ADAR2BlacZ line. Resulting heterozygous mice for the KO and also Cre-negative, were used to maintain the colony by breeding heterozygous by heterozygous. Homozygotes possess a non-functional ADAR3 protein and were thus referred to here as *Adar3*^*exon*3^.

### Histology

Mice were deeply anesthetized and perfused transcardially with 4% paraformaldehyde (PFA) in PBS. Brains were post-fixed in 4% PFA (in PBS) overnight and then cryoprotected in 30% sucrose. Tissue was cryosectioned into 40 μm thick coronal slices and stored at 4°C in PBS + 0.02% sodium azide. Free-floating sections were mounted onto gelatin-coated slides and air-dried prior to cresyl violet staining in a 0.1% solution (ProSciTech, QLD, Australia) according to standard protocols.

### Behavioral testing

The following testing order was performed on all mice: open field testing in the morning of Day 1; elevated plus maze in the afternoon of Day 1; rotarod test 1 on Day 2; rotarod test 2 on Day 3; rotarod test 3 on Day 4; and Y-maze testing on Day 5. Animals used in fear conditioning experiments were not tested in any other behavioral test.

### Open field test

Motor activity and anxiety were evaluated in the open field test (OFT). Mice were placed in the center of the OFT arena (40 × 40 cm; Med Associates, Georgia, VT, USA), which was a large box with clear Plexiglas walls, no ceiling, and a white floor. Each chamber was set inside a larger sound-attenuating cubicle and with two ceiling mounted house lights at the rear corners to fully illuminate the cubicle. A small fan mounted at the top of the right wall of each chamber provided background noise. Testing chambers were cleaned with 70% ethanol (EtOH) before each run to prevent the transfer of odors between animals. Mice were allowed to freely explore the test box for 10 min while a computer software program (Activity Monitor, Med Associates, Georgia, VT, USA) recorded activity via photo-beam detection inside the testing chambers. Data collected includes total distance traveled, distance traveled in the center and periphery of test box, and time spent in the center and periphery.

### Elevated plus maze

Following the OFT, anxiety-like behavior was assessed using the elevated plus maze (EPM). The EPM apparatus (Med Associates, Georgia, VT, USA) used in this study consisted of two platforms (77 × 10 cm) opposite each other and two platforms (77 × 10 cm) enclosed by three 20 cm high walls opposite each other so that they form a plus shape. The maze was elevated 70 cm off the floor. Mice were placed at the center of the maze facing one of the closed arms and allowed to explore freely for 5 min. Each test was video recorded and anxiety-like behavior including the number of entries into each arm and time spent in each arm were measured. The maze was wiped cleaned with 70% EtOH and dried between each mouse.

### Rotarod

Mice were placed on the suspended beam of the rotarod facing away from the viewer for 5 min. The rotarod was started once all mice were placed on the beams and rotated at a rate of 4 rpm and increased to 40 rpm over the course of 5 min. Animals were taken off the rotarod once they fell to the catch tray below or after 5 min had elapsed and the latency to fall off the beam was recorded. Animals were exposed to the test once a day for three consecutive days. The device was cleaned with 70% EtOH between each mouse.

### Y-maze

The Y-maze was adapted from a previously described protocol (Wolf et al., 2016), with slight modification. Testing was conducted in an opaque plexiglas Y-maze consisting of three arms (40 × 4 × 17 cm high) diverging at a 120° angle. Each mouse was placed in the center of the Y-maze and allowed to explore freely through the maze during a 5-min session. The experimenter remained in the room but was not visible to the test mouse. The sequence and total number of arms entered was video recorded. Arm entry was counted when the hind paws of the mouse had been completely placed in the arm. The percent alternation was calculated as the number of trials containing entries into all three arms divided by the maximum possible alternations (the total number of arms entered minus 2) × 100. The maze was cleaned with 70% EtOH and dried between each mouse.

### Contextual fear conditioning experiments

In contextual fear conditioning, animals learn to associate an aversive foot shock (unconditional stimulus; US) with the neutral context in which it was elicited (conditional stimulus; CS). This learning is thought to lead to the formation of a CS-US associative memory which, when retrieved through re-exposure to the context in which conditioning occurred, leads to expression of conditional fear (displayed as freezing behavior in mice). This fear expression is a direct correlate of contextual memory (Maren et al., 2013).

On day one of fear conditioning, mice were placed into a fear-conditioning chamber (32 × 27 × 26 cm; Med Associates, Georgia, VT, USA). The chamber had aluminum sides and clear plexiglas back, top, and front walls, with the front being a hinged door. The floor of the chambers consisted of a metal grid with 36 stainless steel rods spaced 8 mm apart and connected to a current generator for delivery of an electric foot shock. There was also a removable metal tray underneath the grid. Attached to the left wall of each chamber was a small house light measuring 3.8 cm^2^ containing a light bulb that emitted 780 lux at a distance of 2.5 cm and covered by a diffuser. The foot-shock and house light were controlled by a computer software program (FreezeFrame, Med Associates, Georgia, VT, USA). For all fear conditioning experiments, the chambers were setup with the following parameters: (1) chambers were cleaned with 70% EtOH and the grid floor dried; (2) the tray was scented with aniseed essence; and (3) the test room lighting was set at full fluorescence.

On conditioning day, mice were placed into the chambers and after a placement-to-shock interval (PSI) of 3 min a single 2 s 1.0 mA foot shock was delivered. Mice were removed from the chambers 30 s after the shock ended and returned to their home cages. For short-term contextual memory assessment, animals were placed back into the conditioning context 2 h post-conditioning for a 3 min test in which no foot shock was elicited. Upon completion of the context test, mice were removed from the fear conditioning chambers and placed back into their home cages. A separate cohort of animals was used for long-term and remote contextual memory assessment. Animals were conditioned as described above. At 24 h post-conditioning, mice were placed back into the conditioning context for a 3 min test in which no foot shock was elicited. Upon completion of the context test, mice were removed from the fear conditioning chambers and placed back into their home cages. To test remote contextual memory, the same animals were re-tested in the conditioning context for 3 min at 3 weeks post-conditioning.

All fear conditioning experiments were video-recorded and the conditioned fear responses measured by using the number of times the animal was determined to be freezing by observing it every 4 s over the length of the testing period and converting that number into a percentage of time spent freezing over the total time. Freezing behavior is characterized by the cessation of movement except that needed for respiration (Fanselow and Poulos, 2005). Freezing was scored by one investigator, who was blind to the treatment groups.

### RNA extraction

Three wild type and three *Adar3*^*exon*3^ mice were used for RNA-seq experiments. Briefly, each individual was deeply anesthetized before harvesting and dissecting the hippocampal tissue. Total RNA was extracted from mouse hippocampus, using the AllPrep DNA/RNA Mini Kit (QIAGEN, Hilden, Germany) with an on-column DNAse treatment step (to remove genomic DNA), following the manufacturer's instructions. Mouse tissue was homogenized using the Qiagen TissueLyser II according to the manufacturer's instructions. Isolated RNA quality (integrity) was assessed on an Agilent RNA 6000 Nano Chip (Agilent Technologies, Santa Clara, CA, USA) using the Agilent 2100 BioAnalyser (Agilent Technologies, Santa Clara, CA, USA), following the BioAnalyser instructions. RNA concentration was quantified using NanoDrop 2000TM Spectrophotometer (Thermo Scientific, Waltham, MA).

### Library preparation and RNAseq

Libraries were prepared with TruSeq Stranded Total RNA Library Prep Kit with Ribo-Zero (Illumina, San Diego, CA, USA). Libraries were quantified with Qubit™ 3.0 Fluorometer dsDNA HS kit (ThermoFisher Scientific Inc., Waltham, MA, USA). Indexed DNA libraries were analyzed individually using an Agilent Technologies 2100 Bioanalyzer with the DNA 1000 kit (Agilent Technologies, Santa Clara, CA, USA). Libraries were diluted and pooled to a final concentration of 10 nM. Pooled libraries were quantitated with Qubit™ 3.0 Fluorometer dsDNA HS kit (ThermoFisher Scientific Inc., Waltham, MA, USA). RNA sequencing was performed using Illumina HiSeq 2500 System with 100 bp paired-end sequencing.

### Analysis of RNA editing

Additional description of the Bioinformatics analysis approach is included in Data Sheet 1 and Data Sheet 2. Briefly, trimmed paired-end RNA-seq reads were aligned with bowtie v1.1.0 (http://bowtie-bio.sourceforge.net/index.shtml) (Langmead et al., 2009) to the mouse reference genome (mm10, Genome Reference Consortium GRCm38). A-to-G mismatches were called with Samtools v1.12 (http://www.htslib.org/doc/samtools.html) (Li et al., 2009). The positions of common SNPs from dbSNPs138 were removed.

Only positions covered by at least 20 reads with the A-to-G mismatch found on average 5% of the reads were retained. Candidate editing sites were inspected for Strand Bias, Variant Distance Bias and Read Position Bias (Supplementary Methods). To estimate the significance of A-to-I editing level changes with age, we fitted the changes of A-to-G substitution frequency for the 282 filtered high-confidence candidate editing sites (Data Sheet 2) using the logistic regression model. The logistic regression model (function glm in R, v3.2.3) was used to fit the A-to-G substitution frequency for the 282 filtered high-confidence candidate-editing sites with genotype as a predictor. Analysis of variance (ANOVA) was used to obtain which edited sites showed significant change with genotype, applying a false discovery rate threshold of 5%.

### Differential gene expression analysis

Trimmed paired-end reads were aligned against assembly GRCm38.p4 of the mouse genome with STAR v2.5.1a (Dobin et al., 2013) using a pre-built index based on GENCODE m10. Statistics and quality control of the alignment was performed with RSeQC v2.6.1 (Wang et al., 2012). Quantification of the aligned reads was performed with RSEM v1.2.26 (Li and Dewey, 2011). Genes with low counts were filtered out and only genes with 10 counts in at least 2 samples (6 in total) were considered in the downstream analysis in R v3.2.3 (Retrieved from http://www.R-project.org).

Transcript normalization was performed using the trimmed mean of M values (TMM) applied through the edgeR package v3.14 (Robinson et al., 2010). An identifier was called significant if its false discovery rate is below 0.01 after Benjamini-Hochberg correction. The differentially expressed transcripts are additionally annotated through the BioMart portal (http://www.biomart.org/).

### Targeted deep re-sequencing

Candidate editing sites found to have statistically significant differences in editing levels between the WT and *Adar3*^*exon*3^ (9 loci containing 10 editing sites), along with three additional well-characterized editing targets (serotonin receptor *5ht2cr, Blcap* and *Gabra3*) were selected for targeted re-sequencing and were amplified using PCR.

Briefly, 1 μg RNA was used to synthesize cDNA with the SuperScript^TM^ IV First-Strand Synthesis System for RT-PCR kit (Invitrogen, Carlsbad, CA, USA). PCR was performed in a multi-well plate using one primer pair per sample (mouse), the primer sequences and the PCR conditions used for the PCR reactions are detailed in Supplementary Table 2. PCR products were electrophoresed on a 3% agarose gel, to confirm expected product size and the absence of additional bands. All amplicons were quantitated individually using a Qubit™ 3.0 Fluorometer dsDNA HS kit (ThermoFisher Scientific Inc., Waltham, MA, USA). Subsequently the nM concentration for each amplicon was calculated. The 12 PCR amplicons for each sample (mouse) were pooled in equimolar ratios. The pooled PCR products were purified using a MinElute PCR Purification Kit (QIAGEN, Hilden, Germany). 200 ng of each pool was used for library preparation with NEBNext Ultra II DNA Library Prep Kit for Illumina (New England Biolabs Inc, Ipswich, MA, USA; E7645S) with the NEBNext Multioplex Oligos for Illumina (Index Primers Set 1; E7335S) according to the manufacturer's instructions.

The quality and quantification control was performed with a LabChipGX (Perkin Elmer, Waltham, MA, USA) and the KAPA Library Quantification Kit (KAPA Biosystems, Wilmington, MA, USA) with the ViiA7TM Real-Time PCR System (ThermoFisher Scientific Inc., Waltham, MA, USA). 7pM library plus 10% PhiX spike-in control were loaded on MiSeq System (Illumina, San Diego, CA, USA) using the MiSeq Reagent Kit v2, 300 Cycles (Illumina). RNA editing analysis was performed on both paired and unpaired reads mapped to the mouse genome (mm10, Genome Reference Consortium GRCm38) with bowtie1 v1.1.0 (http://bowtie-bio.sourceforge.net/index.shtml) as described above. All potential editing sites were manually inspected using the Integrative Genomics Viewer (IGV v2.3, http://software.broadinstitute.org/software/igv/) (Robinson et al., 2011). A-to-G mismatches in high-quality score bases with frequency of 1% or greater were recorded (Data Sheet 2) and analyzed as shown in Supplementary Figure 2).

### Serotonin receptor editing analysis

The serotonin receptor 5HT2CR is known to be edited on 5 closely positioned adenosines on exon 5, i.e., **A**T**A**CGT**AA**TCCT**A** (highlighted in bold). The editing of all or any combination of these adenosines can result in 32 mRNA variants. To investigate the editing levels at each adenosine position in exon 5 and all its possible combinations, an in-house script was used to generate all possible combinations of the amplified 178 bp PCR 5HT2CR product (chrX:147169590-147169767). Based on these 32 variants, 32 “artificial serotonin chromosomes” were created (Data Sheet 3) and indexed with bowtie1 v1.1.0. (http://bowtie-bio.sourceforge.net/index.shtml) Reads from the targeted deep re-sequencing experiment were aligned to all possible “artificial serotonin chromosomes” with bowtie1 v1.1.0. Bedtools v2.22.0 (Quinlan and Hall, 2010) was used to count the number of reads aligning to each of the 32 possible “artificial serotonin chromosomes.” Statistical significance was determined using a one-way ANOVA in R v3.2.3.

### qPCR validations

To further validate hits with significant difference between WT and *Adar3*^*exon*3^, qPCR reactions were also performed. Briefly, 200 ng of RNA extracted from the right hippocampus was used for a cDNA synthesis. RNA was converted into cDNA in a 20 μl reaction with the SuperScript^TM^ III First- Strand Synthesis System for RT-PCR kit (Invitrogen, Carlsbad, CA, USA), following the manufacturer's instructions. For qPCR, 3 μl cDNA as obtained above was used (diluted 1:20). Negative controls included a non-template (water) control and a reaction without reverse transcriptase added. Reactions were performed using UPL assays (Roche Applied Science, Basel, Switzerland) with LightCycler 480 Probes Master, on the Roche LightCycler 480 (Roche Applied Science, Basel, Switzerland).

Samples were amplified using the standard qPCR program for the Roche LightCycler 480 program as follows: initial denaturation at 95°C 10 min, followed by 40 cycles of: 95°C for 10 s, 60°C for 30 s and 72°C 5 s. The threshold cycle (Ct) was set using the LightCycler 480 Software v1.5.0. (Roche Applied Science, Basel, Switzerland). Amplification efficiency was determined by including a standard curve of cDNA serial dilutions for each primer set. All tested primers (target and standard house-keeping genes) had comparable amplification efficiency (Supplementary Table 3). Gene expression analysis was performed with the comparative Ct method (Schmittgen et al., 2008). All primer sets used are detailed in Supplementary Table 3. Statistical significance was determined with two-tailed unpaired *t*-test in GraphPad Prism v7.00, (GraphPad Software, La Jolla, CA, USA).

### SH-SY5Y cells, staining, and microscopy

Coverslips were coated with Poly-L-Lysine for 5 min, washed with H_2_O four times, and dried overnight at room temperature. SH-SY5Y neuroblastoma cells (CRL-2266^TM^, ATCC®, Manassas, VA, USA) cells were plated and grown overnight at a density of 2.5 × 10^5^ cells in 500 μl DMEM, 10% FBS. Cells were depolarized by adding KCl at a concentration of 50 mM to the medium for 30 sec, after which the medium was replaced, and cells were incubated for 1, 3, and 6 h before fixing. Cells were fixed in 4% paraformaldehyde/4% sucrose in phosphate-buffered saline (PBS) at room temperature for 15 min and washed three times in PBS. Cells were permeabilized in 0.1% Triton X-100/0.1% Na-Citrate/PBS for 3 min at room temperature and washed three times with PBS. Cells were blocked in 10% FBS/PBS for 1 h at room temperature and incubated overnight at 4°C with primary antibody, (1:100, ADARB2; NBP1-57558, Novus Biologics, CO, USA) in blocking solution. The following day, cells were washed 3 times in PBS and then incubated with secondary antibody (1:500 Abcam; ab6939, Goat pAb to Rb IgG-H&L (Cy3®) pre-adsorbed, CBG, UK) for 90 min at room temperature and washed 4 times in PBS and once in Milli-Q® H_2_O before mounting onto microscopy slides with MP Biomedical immune-fluoro mounting medium. A Zeiss LSM700 confocal microscope was used with 63x oil objective. The settings were: pinhole 34 μm, speed 1.58 μs per pixel, 512 × 512 pixels, 4 times averaging, laser intensity 2, and gain was adjusted on the fly.

### Statistical analysis

The description of specific statistical methods, are described in detail in each relevant section. Briefly, for mouse behavioral experiments unpaired two-tail *t*-tests were calculated using GraphPad Prism v7.0 (GraphPad Software, La Jolla, CA, USA). For qPCR gene expression analysis unpaired *t*-test was estimated in GraphPad Prism v7.0 (GraphPad Software, La Jolla, CA, USA). The A-to-G frequency for the 282-filtered candidate editing sites was fitted with the glm model in R v3.2.3, followed by ANOVA. For deep sequencing experiments one-way ANOVA analysis was performed in R v3.2.3.

### Data availability

Metadata and nucleotide sequences generated and utilized in this work were deposited to NCBI under accessions: **Bioproject** PRJNA434770, **BioSamples**: SAMN08574369, SAMN08574370, SAMN08574371, SAMN08574372, SAMN08574373, SAMN08574374 and **SRA accessions**: SRR6757007, SRR6757008, SRR6757009, SRR6757010, SRR6757011, SRR6757012.

## Results

### Expression analysis of *Adar3*

**Figure 1:**
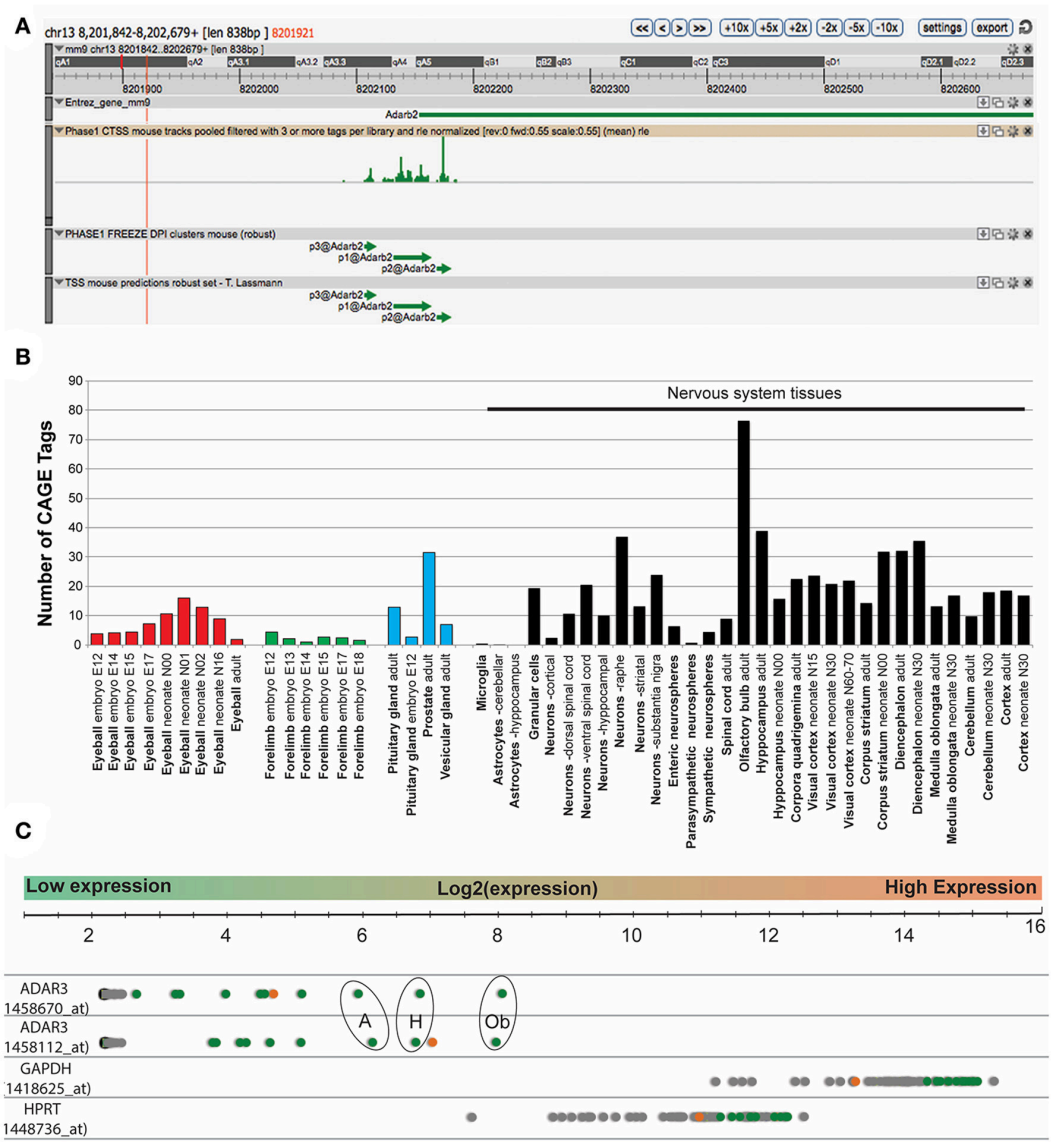
ADAR3 is most highly expressed in mouse nervous tissue. **(A)** Screen shot from ZENBU Genome Browser showing the ADAR3 (*Adarb2*) promoter region (mm9 - chr13:8201842-8202679). Expression analysis of ADAR3, derived from the FANTOM5 project, which shows transcriptional activity measured by Cap Analysis of Gene Expression (CAGE), shows the sense strand Tag expression of a ~838 bp region that surrounds the *Adar*2b gene promoter. **(B)** Out of the 366-mouse tissue samples analyzed from the FANTOM5 project, ADAR3 CAGE tags showed expression above background levels in 51 tissues. Importantly, the majority of these highly expressed tags are from neuronal tissues, with exceptions being the eyeball (red bars), embryonic forelimb (green bars), pituitary gland and prostate (blue bars). **(C)** Similarly, *in-silico* analysis of microarray data obtained from the BioGPS database (http://biogps.org) for ADAR3 expression in 53 mouse tissues using two different probes, shows highest expression in brain. Different brain regions are indicated as green dots, with highest expression in olfactory bulb [Ob], hippocampus [H] and amygdala [A]. The retina is indicated as an orange dot and all other tissues are gray. Two highly expressed housekeeping genes *Gapdh* and *Hptr* are shown for comparison.

In mouse, the *Adar3* gene is located on chromosome 13, with the gene spanning over ~557 Kbp (Figure 1A). We performed an expression analysis of ADAR3 that interrogated publicly available datasets derived from different technologies to exclude any bias. Firstly, data from the FANTOM5 project, which shows transcriptional activity as measured by Cap Analysis of Gene Expression (CAGE), showed Tags produced from a ~838 bp region that surrounds the *Adar*3 promoter (Figure 1A). Out of the 366 mouse tissue samples analyzed, ADAR3 CAGE tags showed expression in 51 tissues above background levels (Figure 1B). Importantly, this analysis highlighted that the majority of these highly expressed tags (indicating high ADAR3 transcription) are from neuronal tissues, with exceptions being the eyeball (red bars), embryonic forelimb (green bars), pituitary gland and prostate (blue bars). Secondly, we performed in-silico analysis of microarray data obtained from the BioGPS database of gene expression across 53 mouse tissues (http://biogps.org). Both ADAR3 probes in this dataset showed that ADAR3 was most highly expressed in the adult mouse brain, with highest expression observed in hippocampus, thalamus, amygdala and olfactory region (Figure 1C). This expression pattern suggests that *Adar3* may be involved in the function of the brain and sensory information processing, i.e., learning and memory formation.

### *Adar3*^*exon*3^ mice are grossly normal

**Figure 2:**
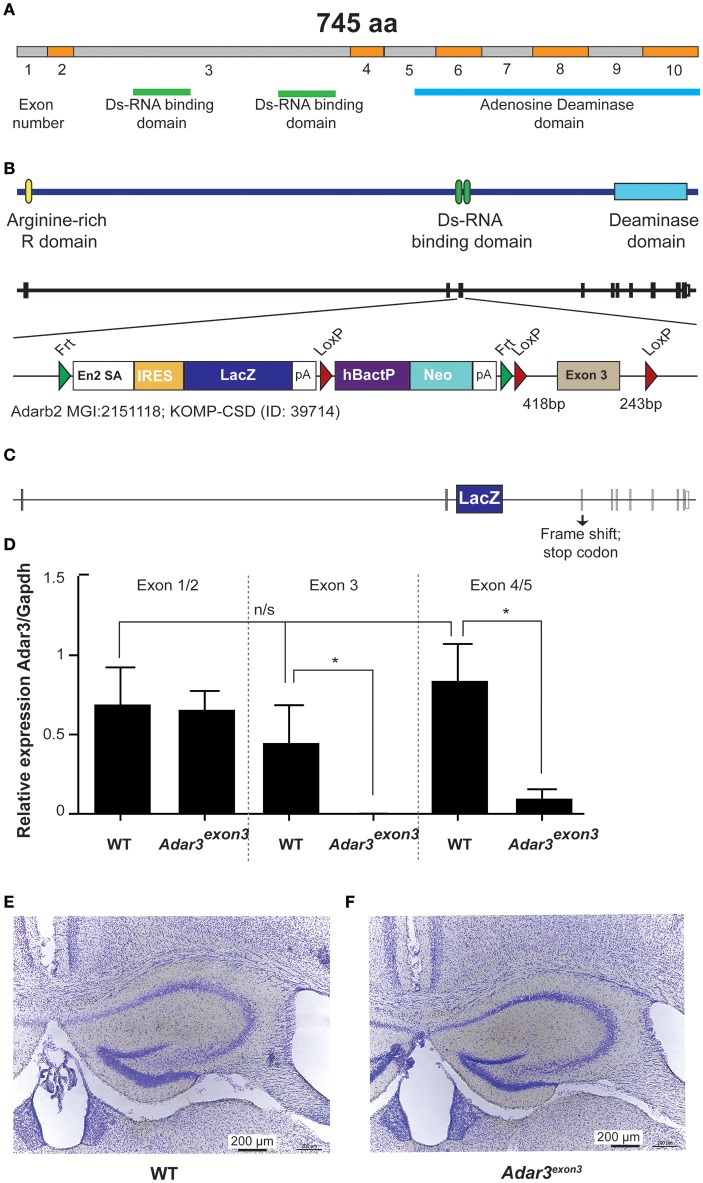
Generation of *Adar3*^*exon*3^mice. **(A)** Schematic representation of *Adar2b* spliced mRNA structure with exons numbered and with the Ds-RNA binding and the Adenosine Deaminase domains shown. **(B)** Schematic of *Adar3* knock-out allele. *Adar3* intronic (lines) and exonic (bars) sequences are drawn to scale. **(C)** Schematic representation of the strategy used to remove exon 3 (containing the Ds-RNA binding domains). **(D)** Relative expression of *Adar3* with respect to *Gapdh*, the relative mRNA level analysis was done by the ΔΔCt method (primers used were as follow 5′-3′: JM371_mADAR3_Ex1/2_f: GAGGTCCAAGAGGAAAGACA; JM372_mADAR3_Ex1/2_r: AGGTTATCTTCATCCTCTGTG; JM373_mADAR3_Ex3_r: TTCACTTCAGCACTGCTGGT; JM374_mADAR3_Ex4/5_f: TCGTCATGACCAAAGGCTTG). ^*^Indicates *P* < 0.05; **(E,F)** No gross differences in the hippocampal formation between the WT and *Adar3*^*exon*3^ mice were found.

In order to test whether ADAR3 is involved in learning and memory, we generated a knockout mouse line, using the Cre/lox gene targeting system. The newly created line lacks exon 3 of the *Adarb2* gene, which encodes two double stranded RNA binding domains (Figures 2A–C). By removing the ability of ADAR3 to bind double stranded RNA, any adverse effects on the RNA editing-dependent and independent functions can be investigated. Quantitative PCR verified that exon 3 was absent in homozygous mice and showed that the level of the (truncated) transcript was decreased (Figure 2D). *Adar3*^*exon*3^ mice were born following the predicted Mendelian ratio and displayed no size or weight difference nor any atypical developmental characteristics compared with controls. As ADAR3 is expressed almost exclusively in the brain we investigated brain morphology in adult mice. *Adar3*^*exon*3^ mice showed no obvious differences in brain morphology including the hippocampal formation (Figures 2E,F).

### *Adar*^*exon*3^ mice display deficits in anxiety and learning

**Figure 3:**
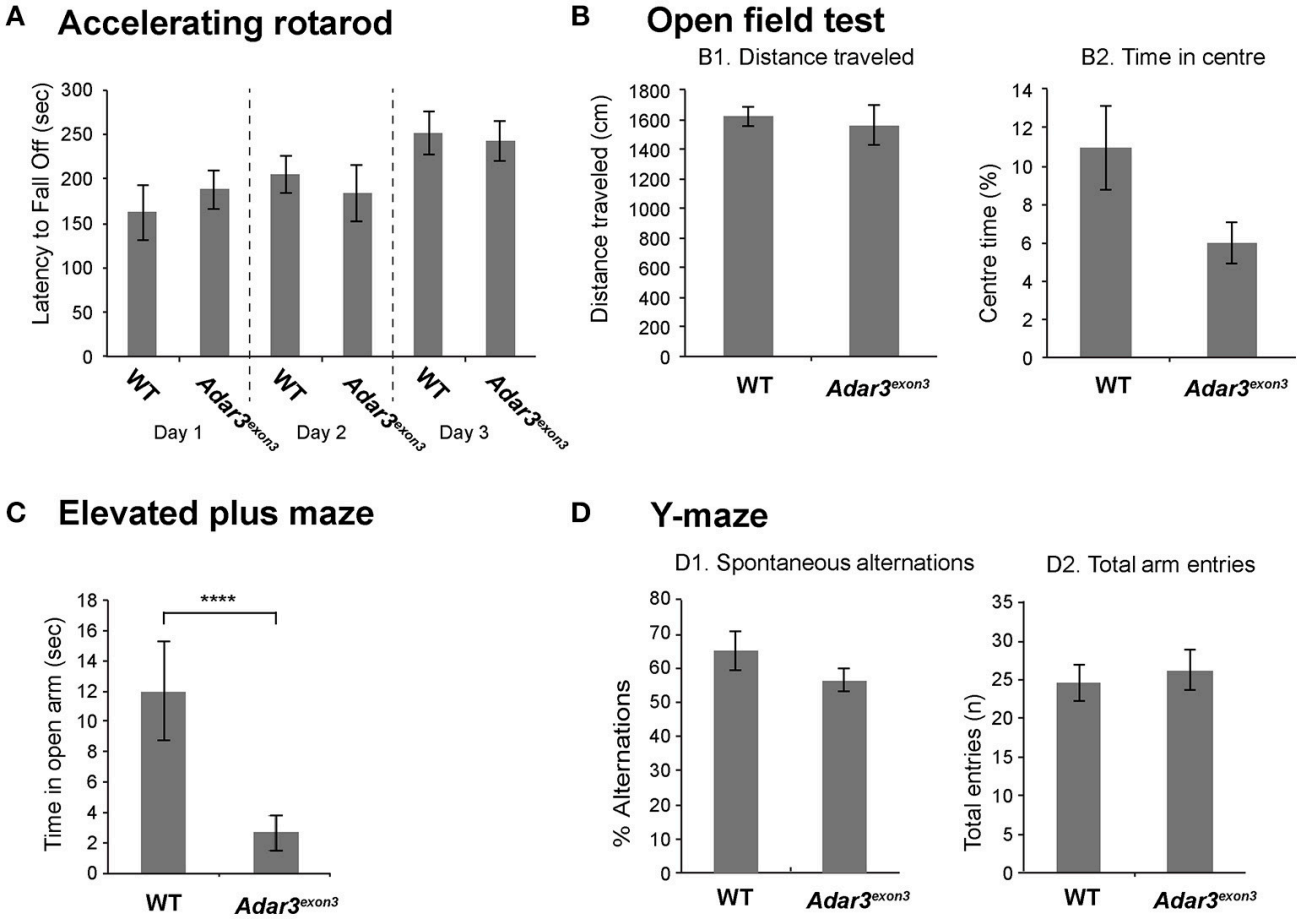
*Adar3*^*exon*3^ mice show increased anxiety in the elevated plus maze test**. (A)** No differences were observed between *Adar3*^*exon*3^ (*n* = 10) and WT littermates (*n* = 11) in the accelerating rotarod over a 3 day testing period indicating normal motor coordination and balance skills. **(B)** *Adar3*^*exon*3^ mice displayed normal locomotor **(B1)** and anxiety **(B2)** behavior as neither the total distance traveled nor the time spent in the center of an open field test were statistically significant between *Adar3*^*exon*3^ (*n* = 10) and WT littermates (*n* = 11). **(C)** However, in the elevated plus maze test of anxiety, *Adar3*^*exon*3^ (*n* = 10) mice spent significantly less time in the open arms as compared to WT littermates (*n* = 11) indicating an anxiogenic phenotype in the *Adar3*^*exon*3^ mice. **(D)** No significant differences were found between *Adar3*^*exon*3^ (*n* = 10) and WT littermates (*n* = 10) in either the percentage of spontaneous alternations **(D1)** or total arm entries **(D2)** in the Y-maze suggesting normal spatial learning and memory. Error bars indicate SEM. Star (***) indicates *P* < 0.05.

Taking into account that *Adar3*^*exon*3^ mice are grossly normal, and that the function of *Adar3* in mice is more likely to be cognitively subtle, we proceeded with behavioral analyzes. Locomotor activity in *Adar3*^*exon*3^ mice was normal over three consecutive days of testing using the rotarod test (Figure 3A). As ADAR3 is highly expressed in the hippocampus we sought to test *Adar3*^*exon*3^ mice using hippocampal-dependent behavioral analyzes. Firstly, we employed the open field test to investigate locomotor activity (Figure 3B1) and anxiety (Figure 3B2) and found no significant differences between control and *Adar3*^*exon*3^ mice, although there was a trend toward decreased anxiety in *Adar3*^*exon*3^ mice (*p* = 0.0633).

In contrast, in an alternate test for anxiety-like behavior, *Adar3*^*exon*3^ mice subjected to the elevated plus maze showed significant differences in anxiety suggesting impairment (Figure 3C) and potentially reflecting alterations in some forms of hippocampal processing (Bannerman et al., 2014). Finally, we used the Y maze test, which is a spatial working memory test that measures the willingness of mice to explore new environments. We found no significant differences between *Adar3*^*exon*3^ and control mice in percentage alternations (Figure 3D1) or total arm entries (Figure 3D2).

**Figure 4:**
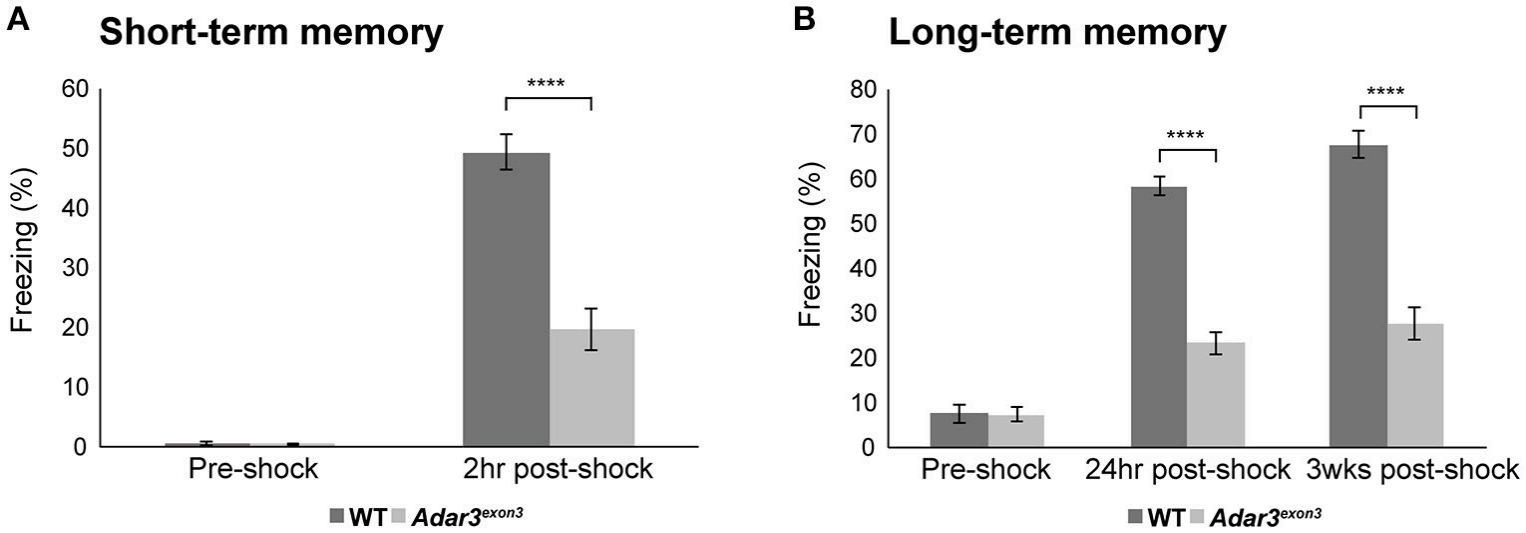
Impaired hippocampal-dependent context fear learning in *Adar3*^*exon*3^ mice. **(A)** Short-term memory. *Adar3*^*exon*3^ mice displayed significantly reduced freezing behavior compared to WT littermates when tested 2 h after a mild foot-shock in a contextual fear-conditioning task (*n* = 10/genotype). **(B)** Long-term and remote memory. *Adar3*^*exon*3^ animals showed significantly impaired freezing behavior when tested both 24 h and 3 weeks post-conditioning as compared to WT littermates (*n* = 10/genotype). In both tests, all groups displayed minimal freezing behavior prior to the foot-shock being delivered indicating no alterations to baseline fear levels in either genotype. Error bars indicate SEM. Star (****) indicates *P* < 0.0001.

The hippocampus and amygdala, where ADAR3 expression is high (Figure 1C) are also central in fear conditioning and the hippocampus is implicated in the formation of context-dependent memory while the amygdala coordinates the paired association of the stimuli resulting in a fear response (Selden et al., 1991). Therefore, we used a classical fear conditioning experiment to test the contextual memory of *Adar3*^*exon*3^ mice. In this experiment mice are placed in a novel environment (fear conditioning chamber), where a foot shock is delivered. Mice learn to associate the contextual cues of the chamber with the aversive stimulus (see Methods). Re-exposure to the conditioning context retrieves the fear memory (displayed as freezing behavior). For short-term memory we re-exposed mice to the context (fear conditioning chamber) 2 h after the first shock was delivered. We found robust learning in control mice and, while some learning was observed in *Adar3*^*exon*3^ mice, the response was significantly decreased (Figure 4A). These results were mirrored in both long-term (24 h post-shock; Figure 4B) and remote memory after 3 weeks post-shock; Figure 4B) tests. These tests showed that *Adar3*^*exon*3^ mice display an anxiogenic-like phenotype in the elevated plus maze test and impaired contextual fear learning and memory.

### *Adar3* deficiency results in a subtle modulation of hippocampal gene expression

As the hippocampus is essential in contextual fear conditioning (Frankland et al., 1998; Maren et al., 2013) and the ventral hippocampus has been shown to play a role in the mechanisms of stress and anxiety (Bannerman et al., 2003, 2014), we reasoned that ADAR3 deficiency may affect hippocampal function. In order to assess how ADAR3 deficiency modulates gene expression in the hippocampus we performed next generation sequencing on total RNA extracted from hippocampal tissue of WT and *Adar3*^*exon*3^ mice. Total RNA from the hippocampus was sequenced and differential gene expression analysis was performed (see Methods and Supplementary Table 1).

**Figure 5:**
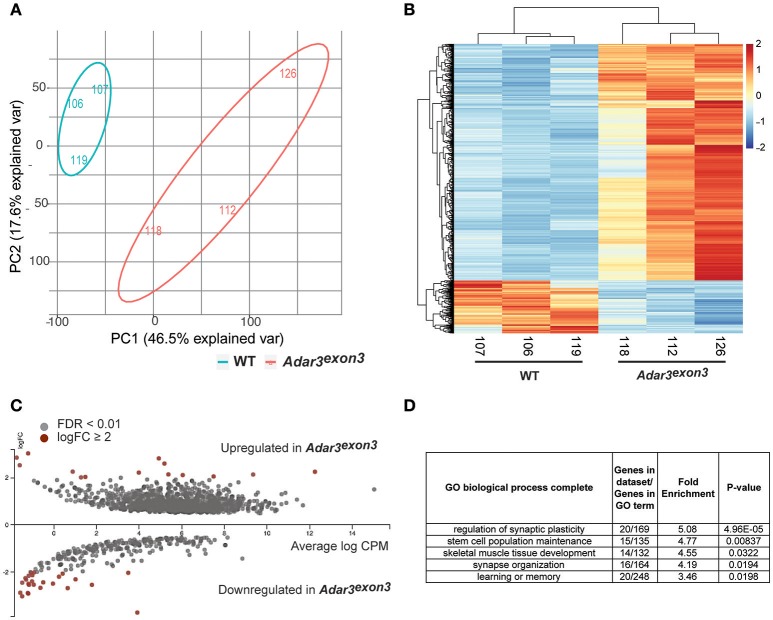
*Adar3* deficiency results in a mild modulation of gene expression in the hippocampus. **(A)** A Principal Component Analysis of normalized gene counts showing the transcriptomic variation between the samples in two dimensions. Most of the variation could be explained by PC1 and PC2. **(B)** A heat map of z-scores of differentially expressed genes (FDR < 0.01) of WT and *Adar3*^*exon*3^ mice. X/Y axes are ordered based on unsupervised clustering. **(C)** An MA plot of differentially expressed genes with FDR <0.01. Genes with log2FC≥ 2 are highlighted in red. Image generated with Degust, v.0.20 (http://vicbioinformatics.com/degust/index.html). **(D)** Panther gene ontology (GO) analysis of 550 (508 with mapped IDs) differentially expressed genes with FDR <0.01, log2FC≥ 1). Analysis Type: PANTHER Overrepresentation Test (release 20160715), GO Ontology database version 1.2, released 2017-01-26 (Ashburner et al., 2000; Mi et al., 2013, 2017).

Principal component analysis (herein, PCA) separated WT from *Adar3*^*exon*3^ samples (Figures 5A,B). A total of 550 differentially expressed genes (DEGs) were identified at a false discovery rate (FDR) of <0.01 and a logFC of at least 1. The majority of DEGs were up-regulated in the *Adar3*^*exon*3^ hippocampi (423), while 127 genes were down-regulated (apart from *Adar2b*), as shown in Figures 5A,B. The results from the DEG analysis were validated for 16 transcripts using quantitative polymerase chain reaction on an independent cohort of RNA samples (Supplementary Figure 1). *Sipa1l3, Kcnc3, Dagla, Pgbd1*, and *Dkkl1* genes, showed statistically significant changes in gene expression in agreement with the RNA-seq analysis, while the majority of the rest of the tested genes showed the expected trend but did not reach significance.

Overall, most of the differentially regulated genes (with statistical significance), showed subtle fold changes in gene expression, with just a few genes with logFC ≥ 2 (Figure 5C). Gene ontology analysis of the DEGs with logFC ≥ 1 showed enrichment for biological processes such as regulation of synaptic plasticity and stem cell population maintenance (Figure 5D). Moreover, Ingenuity Pathway Analysis (IPA) showed that the most significantly affected pathways from the DEG analysis are all pathways related to the nervous system and oncogenesis, *i.e.*, Rac Signaling, Axonal Guidance Signaling, Glutamate Receptor Signaling and Wnt/β-catenin Signaling (Data Sheet 4).

**Figure 6:**
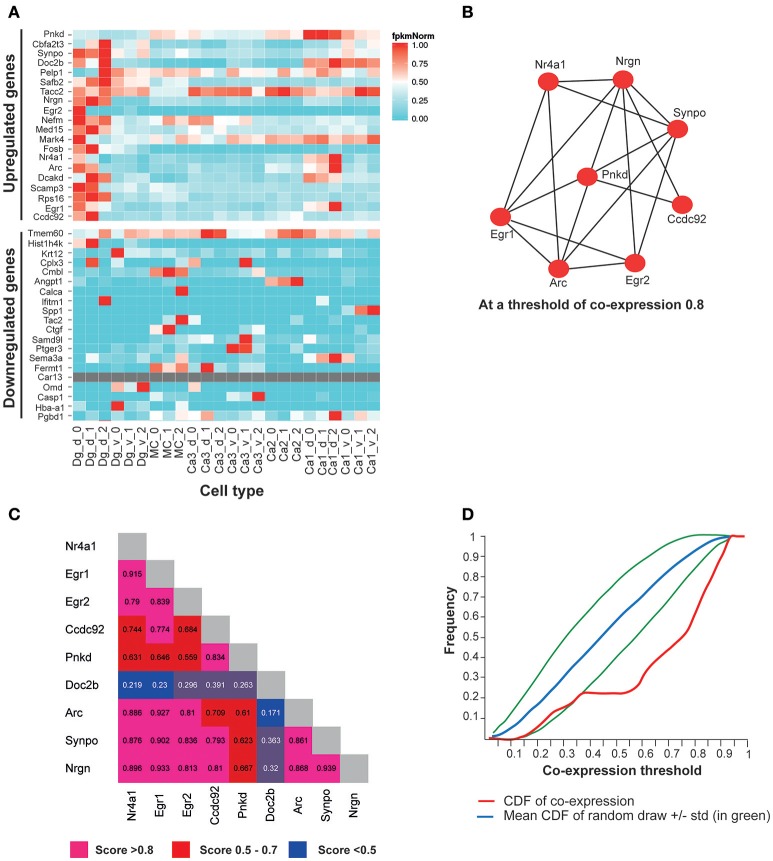
The top most significantly overexpressed genes in the *Adar3*^*exon*3^ hippocampi show signature of co-expression. **(A)** A heatmap of FPKM expression values for the 20 most significantly up-regulated genes in the *Adar3*^*exon*3^ hippocampi with entries in the Hipposeq database. Computed from the Hipposeq Web portal (http://hipposeq.janelia.org), replicates included (numbers 0, 1, and 2 on the x axis), normalized values (FPKM values are normalized to the highest expressing population of each gene). **(B)** A co-expression network of a subset of 9 genes (the 20 most significantly up-regulated genes in the in the *Adar3*^*exon*3^ hippocampi with entries in the mouse brain architecture database http://addiction.brainarchitecture.org). **(C)** Adjacency matrix for **(B)**, numbers denote cosine similarity values. **(D)** Cumulative distribution function (CDF) for the subset of 9 genes from **(B)** (in red). The red curve sits below the mean CDF of random draws of genes (in blue) (simulated set). Therefore the subset of 9 genes appears to be more co-expressed than expected by chance. Legend: dg-d, dorsal dentate gyrus granule cell, dg-v, ventral dentate gyrus granule cell, ca1-d, dorsal CA1 pyramidal cell, ca1-v, ventral CA1 pyramidal cell, ca3-d, dorsal CA3 pyramidal cell, ca3-v, ventral CA3 pyramidal cell, mc, dorsal dentate gyrus mossy cell, ca2, dorsal CA2 pyramidal cell.

We also analyzed the sub-hippocampal expression pattern of the most significantly up/downregulated genes by FDR rate, using a recently published public gene expression resource from excitatory neuron-populations in sub-hippocampal regions (http://hipposeq.janelia.org) (Cembrowski et al., 2016). Interestingly, the 20 most significantly upregulated genes in the hippocampi of *Adar3*^*exon*3^ mice, were co-expressed in the dorsal dentate gyrus, whereas the 20 most significantly down-regulated genes did not show a co-expression pattern in any particular region (Figure 6A). Furthermore, in order to determine if the subset of up-regulated genes form a co-expression network brain-wide, we utilized the brain architecture database (http://addiction.brainarchitecture.org/) with 3,041 genes available for mouse, whose expression energies have the highest correlation between the coronal and sagittal images from the Allen Mouse Brain Atlas database brain-wide (available from: mouse.brain-map.org) (Lein et al., 2007; Ng et al., 2009; Hawrylycz et al., 2011a,b).

In the brain architecture database, a gene-by-gene co-expression matrix is computed for the 3,041 genes as described in Menashe et al., Equation 1 (Menashe et al., 2013). The more co-expressed any pair of genes are, the closer their cosine similarity is to 1. The results showed a total of 9 genes were found in the intersection of the 20 most significantly upregulated genes in *Adar3*^*exon*3^ mice and the available genes in the mouse brain architecture database. The 9 genes showed a significant degree of co-expression in the mouse brain (Figures 6B,C), with cosine similarity threshold of 0.8 connecting 8 of the 9 genes in a network (Figure 6B). To determine whether the subset of up-regulated genes is more co-expressed than expected by chance, the cumulative distribution function (CDF) of the entries of the 9 genes and the mean CDF resulting from random draws of genes were computed through the Brain Architecture Web portal (Figure 6D). As the red curve of the CDF (subset of 9 genes) sits below the simulated average of the CDF of random sets of genes, the results suggest that the 9-gene subset may be co-expressed at a higher level than that expected by chance (blue and green lines; Figure 6D). The most significantly down-regulated genes showed less co-expression than expected by chance (data not shown).

### *Adar3* deficiency does not substantially modulate RNA editing activity

ADAR3 has been thought to play a regulatory role by inhibiting the editing activities of ADAR1 and ADAR2 (Chen et al., 2000). In these studies, human purified recombinant ADAR3 lacked editing activity against well-known edited substrates (*GluR-B* and *5-HT2CR* human gene transcript fragments) *in vitro*. In addition, ADAR3 inhibited the site-selective editing of ADAR1 and ADAR2 on adenosines situated on the *5-HT2CR* fragment. However, ADAR1 and ADAR2 also interfered with each other's site-selective editing activity (Chen et al., 2000), suggesting that possibly the precise ratio of the three ADARs *in vivo* may be important for a tight regulation of the editing levels of a particular editing site.

Despite the lack of measurable editing activity *in vitro* (Melcher et al., 1996a; Chen et al., 2000), it has been noted that ADAR3 deaminase domain is probably functional based on the sequence comparison with the deaminase domains of ADAR1 and ADAR2. All amino acid residues that are believed to be part of the deaminase domain in ADAR3 apart from a few amino acids are well conserved among the ADAR members (Chen et al., 2000). It has also been shown that unlike the recombinant ADAR3, the native ADAR3 from mouse brain, appears to form a homodimeric-complex and homodimerization may be required for the function of ADAR1 and ADAR2 (Cho et al., 2003). Therefore it has been speculated that ADAR3 activity could be different *in vivo* compared to the *in vitro* studies (Cho et al., 2003).

**Figure 7:**
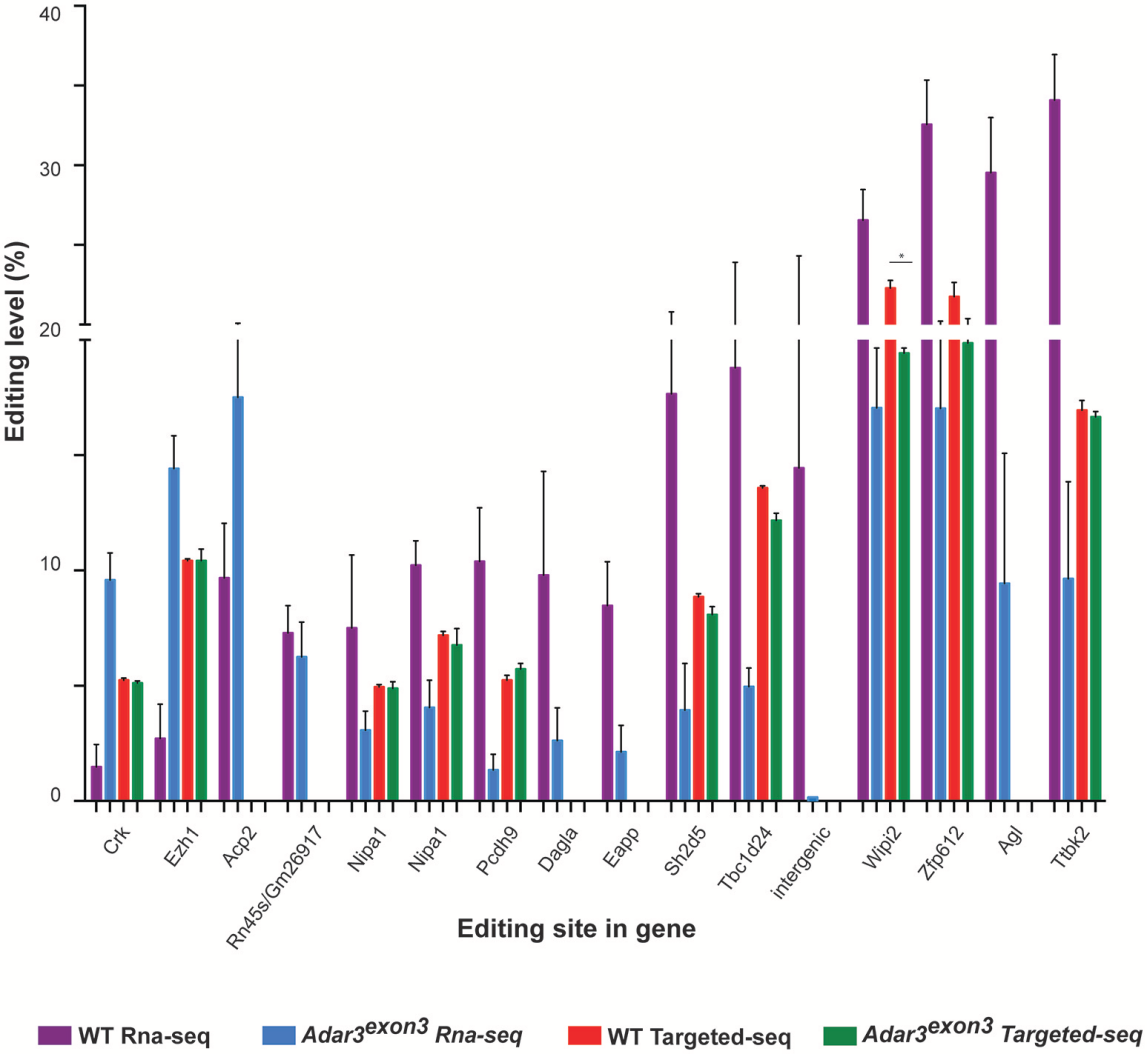
*Adar3* deficiency does not have a substantial impact on RNA editing levels. Editing levels of 16 sites that showed significant differences between WT and *Adar3*^*exon*3^ mice in the RNA-seq analysis (purple and blue bars) compared to the editing level of 10 sites chosen for validation through targeted PCR re-sequencing (red and green bars). (*n* = 3/genotype) Error bars indicate SEM. Star (*) indicates *P* < 0.05.

To investigate whether a measurable alteration in editing activity could be identified in the *Adar3*^*exon*3^ mice, RNA-editing analysis from the mouse hippocampi was performed. The results showed that out of the 282 high-confident editing sites (see Methods, Data Sheet 1 and Data Sheet 2), 52.5% of them known editing targets in the RADAR v2 database (http://rnaedit.com), only 16 genomic positions showed statistically significant differences in editing frequency between the WT and *Adar3*^*exon*3^ mice as summarized in Figure 7 and Supplementary Figure 2. In order to validate these differences, targeted PCR of the locus surrounding selected candidate editing sites was performed, followed by deep sequencing with the Illumina MiSeq system (see Methods and Supplementary Figure 2). Out of the 10 sites chosen for validation, only one site (in the gene *Wipi2*) showed significant difference of more than 2% (2.87 and 4.67% for a neighboring site) between the WT and *Adar3*^*exon*3^ mice (Figure 7).

In addition we chose to assess how *Adar3* deficiency modulates the editing frequency of three protein-coding genes with well studied and characterized editing sites–*Blcap, Gabra3* and serotonin receptor *5-Ht2cr*. Bladder cancer-associated protein (BLCAP), also known as BC10, is a highly conserved 87 Amino Acid protein with an unknown function and multiple editing sites in the coding (Y/C, Q/R, K/R) and non-coding sequences (Gromova et al., 2001, 2002; Clutterbuck et al., 2005). Both ADAR1 and ADAR2 can edit *Blcap* coding sites but with different site-selective efficiencies (Galeano et al., 2010). *Gabra3* codes for the alpha 3 subunit of the GABA(A) receptors, ligand-gated chloride channels and the major inhibitory neurotransmitter receptors in the CNS. *Gabra3* is edited in the coding I/M site with high frequency in the adult brain (close to 100%) (Ohlson et al., 2007). Both ADAR1 and ADAR2 can edit *Gabra3* and the non-edited and edited Gabra3-containing channels show different properties (Rula et al., 2008). The serotonin receptor 5-HT2CR is a G-coupled protein receptor and binds the endogenous excitatory neurotransmitter serotonin. Five adenosines located in close proximity on exon 5 (coding for the intracellular II loop region) are edited and these sites are known as the A, B, C, D, and E sites. Editing of any of these sites or a combination of them has a dramatic effect on the G-protein coupling efficiency of the receptor (Burns et al., 1997; Fitzgerald et al., 1999; Niswender et al., 1999; Wang et al., 2000). ADAR1 selectively edits the A and B site while ADAR2 selectively edits the D site (Nishikura, 2006).

*Adar3* deficiency did not affect significantly any of the above-mentioned well-known editing sites, apart from a significant but small change in the editing of site C of the serotonin receptor as shown in Supplementary Figure 3. The results also showed that although there was a trend for a decrease in the editing frequency of site A and B of the serotonin receptor and the I/M site in *Gabra3*, this was not significant (Supplementary Figure 2).

The deep sequencing of the serotonin receptor exon 5 region also allowed us to study the frequency of the 32 possible mRNA isoforms, resulting from all possible combination of the 5 edited adenosines (Abbas et al., 2010). The largest differences between transcript frequencies were non-significant trend for a decrease in the frequency of the fully unedited transcript (aaaaa), accompanied by an increase in the edited transcripts (aaaag and ggaag) Supplementary Figure 3A). The serotonin transcripts can also be grouped in larger transcript groups (Abbas et al., 2010) and we found a non-significant trend for a decrease in the frequency of the unedited sites in A and B transcript groups with a concurrent increase in the edited (A and B sites) group in the *Adar3*^*exon*3^ animals (Supplementary Figure 3B).

We also examined in detail the editing of the Q/R and the R/G editing sites of the a-amino-3-hydroxy-5-methylisoxazole-4-propionic acid (AMPA) receptor *GluR-B* (*Gria2*) subunit. The Q/R site is known to be edited with close to 100% efficiency almost exclusively by ADAR2 (Sommer et al., 1991; Melcher et al., 1996b). The frequency of editing of the R/G site, which is embedded in the splice donor upstream of the acceptors of the mutually exclusive “flip” and “flop” modules, differs in the two alternative isoforms, whose expression is precisely regulated in sub-hippocampal regions (Sommer et al., 1990; Lomeli et al., 1994). We found no differences in the editing frequency of the Q/R or R/G site in the hippocampi of WT and *Adar3*^*exon*3^ mice (Supplementary Figure 4). While no large differences in editing were found, we also did not find that ADAR3 deficiency results in an increase in the editing frequency of any of the serotonin receptor sites, arguing that unlike *in vitro*, ADAR3 in the hippocampus does not show an inhibitory activity on the editing frequency of any of the known edited *5-Ht2cr* sites *in vivo*.

### Activity-dependent ADAR3 translocation to the nucleus

ADAR3 is known to contain a nuclear import signal where the N terminal R domains bind to the Importin α protein family member KPNA2 (Maas and Gommans, 2009). Furthermore, our data suggests ADAR3 is involved in activity-dependent processes, such as synaptic plasticity, so we reasoned that ADAR3 cellular localization might be influenced by neuronal activity. To check this hypothesis we utilized stimulated SH-SY5Y cells with 50 mM KCl to depolarize the cells in order to mimic neuronal activity.

**Figure 8:**
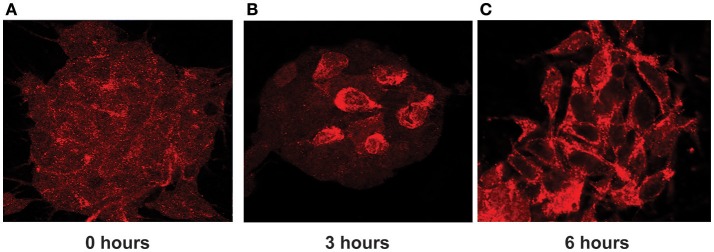
ADAR3 translocates transiently from the cytoplasm to the nucleus upon neuronal activation. **(A)** ADAR3 immunohistochemistry in SH-SY5Y cells shows mostly diffuse ADAR3 staining in the cytoplasm at 0 h. **(B)** At 3 h post KCl-mediated activation, ADAR3 protein shows mostly nuclear localization **(C)** but by 6 h ADAR3 is seen to be returning to a more cytoplasmic pattern.

Indeed, we found that in stimulated cells, ADAR3 was localized to both the cytoplasm and nucleus as shown in Figure 8A. However after 3 h post treatment with KCL, localization of ADAR3 was more evident in the nucleus rather than both (Figure 8B). After 6 h post KCl activation, the ADAR3 protein was markedly diminished in the nucleus (Figure 8C).

## Discussion

In this study we show for the first time that, *Adar3*^*exon*3^ mice are viable, appear to be developmentally normal and are fertile, but exhibit cognitive and behavioral changes, specifically deficits in contextual fear learning and increased anxiety-like behavior; deficits that have been previously linked to the hippocampus (Kheirbek et al., 2013). We also show that both synaptic function-related genes and stem cell maintenance/neurogenesis genes are dysregulated in the hippocampi of *Adar3*^*exon*3^ mice.

In contrast, *Adar1*-deficient mice are embryonically lethal due to unsuppressed global innate immune response (Hartner et al., 2009), while *Adar2*-deficient animals die shortly after birth due to epileptic seizures, caused by deficiency in editing of the Q/R site of *GluR-B* (Brusa et al., 1995; Higuchi et al., 2000). ADAR1 function has been linked to the innate cytoplasmic dsRNA, which can trigger toxic innate immune response (Hartner et al., 2004; Mannion et al., 2014; Liddicoat et al., 2015; Pestal et al., 2015). Apart from editing the essential for life evolutionary conserved Q/R site of the glutamate receptor *GluR-B* subunit, ADAR2 also preferentially edits a number of other sites located on protein-coding regions such as those on the serotonin receptor (Nishikura, 2006). However, the biological function of editing, except for the known cases where editing results in non-synonymous amino acid changes, remains largely unknown. This is because the vast majority of editing occurs on repetitive elements in the UTRs and intronic sequences of mRNAs and in intergenic transcripts (Athanasiadis et al., 2004; Blow et al., 2004; Ramaswami et al., 2012; Huntley et al., 2016). It is unclear how editing in noncoding sequences might influence gene expression in a cis or trans manner. However, possibilities include modulation of miRNA binding sites, regulation of miRNA biogenesis and cross-talk with the short interference pathway (RNAi) (Nishikura, 2006; Liu et al., 2014; Warnefors et al., 2014; Bahn et al., 2015).

RNA-seq analysis of *Adar3*^*exon*3^ mouse hippocampi revealed a modest alteration in the expression of genes enriched for biological processes such as synaptic plasticity and stem cell population maintenance. In the latter there is a partial overlap with genes involved in Wnt/β-catenin signaling, a significantly affected pathway based on the Ingenuity Pathway Analysis in *Adar3*^*exon*3^ mice. The dentate gyrus in the hippocampus is also known to be one of the two places where neurogenesis in the adult brain can occur and the Wnt/β-catenin signaling pathway is primarily involved in regulating adult hippocampal neurogenesis (Lie et al., 2005; Deng et al., 2010). Neurogenesis is also required for memory formation, although its importance in contextual fear conditioning remains controversial (Deng et al., 2010). We also found that the 20 most significantly overexpressed genes in the *Adar3*^*exon*3^ hippocampi seem to form a co-expression network. Interestingly, at least 4 of them, *Fosb, Nrna1, Arc*, and *Egr1*, are known activity-dependent immediate early genes, implicated in synaptic plasticity, whose expression has been associated with learning and memory (Guzowski et al., 2005; Deng et al., 2010; West and Greenberg, 2011; Madabhushi et al., 2015).

The expression of *Nrna1, Arc, Egr1*, and *Egr2* has also been shown to decrease with age in the medial prefrontal cortex in rats (Ianov et al., 2016). These results taken together lead us to hypothesize that *Adar3* deficiency may lead to alteration of genes, involved in synaptic function, cognition and possibly regulation of neurogenesis, which may contribute to the memory and learning deficit phenotype in *Adar3*^*exon*3^ mice, however, the precise molecular mechanism remains unclear. Although the changes in gene expression are subtle, the 20 most significantly overexpressed genes form a co-expression network. Thus, it is possible that the mild modulation of whole networks has implications in the function of the hippocampus, particularly in relation to contextual learning and memory.

While we observed substantial changes in RNA editing for several candidate editing sites in the RNA-seq experiment, targeted PCR re-sequencing of selected target sites revealed only subtle changes in their editing frequencies. It is difficult to determine the biological relevance of such small changes. For example, zebrafish larvae deficient for fragile X mental retardation (fmr1) showed an increase of 1.2–3.7 fold in the expression levels of all ADAR genes (Shamay-Ramot et al., 2015). However, only mild changes in editing were observed in whole larvae, while the changes in editing levels somewhat increased for selected targets when brains were analyzed alone (Shamay-Ramot et al., 2015). Therefore, it is possible that the small and subtle differences found in editing in the *Adar3*^*exon*3^ hippocampi are amplified in specific sub-hippocampal regions.

ADAR3 has been shown to inhibit the editing of the serotonin receptor *5-HT2CR in vitro* (Chen et al., 2000) and we expected to see an up-regulation in the editing frequency of the 5 edited sites on exon 5 of the receptor in *Adar3*^*exon*3^. However, targeted deep sequencing of the receptor revealed a trend for down-regulation of the editing level of 4 of the known edited sites, but no up-regulation was observed for any of the 5 sites. Therefore, unlike *in vitro* experiments, ADAR3 may not be a negative regulator of serotonin receptor editing *in vivo* in the mouse hippocampus. Thus, it is possible that the main biological activity of ADAR3 is exerted during neuronal stimulation. In human cell cultures, stimulated neuroblastoma SH-SY5Y cells showed a transient increase in ADAR3 nuclear localization. Moreover, activity-dependent temporal dynamics in the nuclear concentration of ADAR3 may change the local concentration ratio of the three ADARs and subsequently have a significant impact on gene expression or editing activity.

In our study, we identified that *Adar3* deficiency resulted in the increased expression of genes involved in synaptic signaling and plasticity such as the Potassium Voltage-Gated Channel Subfamily C Member 3 [*Kcnc3* (*Kv3.3*)] and Diacylglycerol Lipase Alpha (*Dagla*). It has been shown that tuning the activity of Kv channels can lead to broadening of the presynaptic action potential (AP) and affecting synaptic transmission (Carta et al., 2014), which in turn would influence the hippocampal CA3-dentate gyrus interaction strength, and thus memory formation (Carta et al., 2014). Interestingly, the modulation of Kv channels can be achieved in a retrograde fashion, by the lipid arachidonic acid, released in post-synaptic hippocampal CA3 pyramidal cells upon activation, leading to a lower threshold to induce long term potentiation on the same post-synaptic CA3 neurons (Carta et al., 2014). Arachidonic acid could be formed from the highly abundant in the CNS endocannabinoid 2-Arachidonoylglycerol (2-AG), synthesized from diacylglycerol almost exclusively by DAGLA (Tanimura et al., 2010). Thus, the potassium channel KCNC3 and DAGLA activities could be interconnected through lipid intermediates (such as arachidonic acid). It remains to be determined how increased expression of *Kcnc3* and *Dagla* in the *Adar3*^*exon*3^ mice modulates synaptic signaling and whether it contributes to the fear conditioning deficit and increased anxiety phenotype in these mice. However, there is mounting evidence to suggest that ion channels (such as potassium channels) are involved in the pathogenesis of a range of psychiatric conditions (Imbrici et al., 2013).

In summary, our results showed that *Adar3*^*exon*3^ mice (lacking exon 3 of the *Adar2b gene*) are viable and appear to be developmentally normal. However, our *Adar*^*exon*3^ mice display cognitive and behavioral changes *i.e.*, have increased levels of anxiety and deficits in hippocampus-dependent short- and long-term memory formation. RNA sequencing experiments from *Adar3*^*exon*3^ mice hippocampi, revealed a dysregulation of genes involved in synaptic function, which results in a subtle modulation of hippocampal gene expression. Moreover, ADAR3 deficiency does not substantially modulate the RNA editing activity of genes known to be edited. Finally, we also show that ADAR3 transiently translocated from the cytoplasm to the nucleus upon KCl-mediated activation in SH-SY5Y cells. *Adar3*^*exon*3^ mice provide an important tool that can be used further to investigate the role and mechanisms of action of ADAR3 in mammalian brain function.

## Ethics statement

This study was carried out in accordance with the recommendations of the Australian code for the care and use of animals for scientific purposes. The protocol was approved by the Garvan/St Vincent's Animal Ethics Committee.

## Author contributions

DM, GB, LK, BG, LA, RZ, MB, NJ, LC, DK, AG, NS, MP, CW, and GF performed the experiments or provided data. DM, GB, LA, BV, and JM conceived the experiments. DM, GB, LK, SP, and JM wrote the manuscript. JM and BV provided laboratory facilities. All authors commented and contributed toward the final version of the manuscript.

### Conflict of interest statement

The authors declare that the research was conducted in the absence of any commercial or financial relationships that could be construed as a potential conflict of interest.
