# Supplementary material for: Adar3 Is Involved in Learning and Memory in Mice
Source: Front Neurosci. 2018 Apr 13;12:243. doi: 10.3389/fnins.2018.00243 (PMC5914295; doi:10.3389/fnins.2018.00243)
Supplement: Supplementary file 1 [file DataSheet1.DOCX]

**Supplementary Material 1**

**Adar3 is involved in learning and memory in mice**

Dessislava Mladenova, Guy Barry, Lyndsey M. Konen, Sandy S. Pineda, Boris Guennewig, Lotta Avesson, Raphael Zinn, Nicole Schonrock, Maina Bitar, Nicky Jonkhout, Lauren Crumlish, Dominik C. Kaczorowski, Andrew Gong, Mark Pinese, Gloria R. Franco, Carl R. Walkley, Bryce Vissel and John S. Mattick*.

***Correspondence: Dr John S. Mattick:** *j.mattick@garvan.org.au*

**Supplementary Methods**

**RNA Editing Analysis**

Adapter and quality trimming was performed with Trim Galore v0.3.7 (http://www.bioinformatics.babraham.ac.uk/projects/trim_galore/) including adapter cutting and a Quality Phred score cutoff of 20. The trimmed reads were mapped with bowtie v1.1.0 (http://bowtie-bio.sourceforge.net/index.shtml) (Langmead et al., 2009) to the mouse reference genome (mm10 –Genome Reference Consortium GRCm38) with main parameters "-n 3 -l 20 -m 1 -e 140 --best". PCR and optical duplications were marked. Properly paired reads aligned to the forward and reverse DNA strand were separated with Samtools v0.1.19. (http://www.htslib.org/doc/samtools.html) (Li et al., 2009). A-to-G mismatches were called with Samtools v0.1.19 and v1.12 mpileup. The positions of common SNPs from dbSNPs138 were removed.

Only positions covered by >20 reads with the A-to-G mismatch found on average 5% of the reads were retained. Positions where more than 3 samples had editing levels of 100% were removed. In addition positions where there were <3 reads with the A-to-G mismatch and any other type of mismatch has been found for the same position were removed. Finally positions where the reads with the reference A or G base is equal or greater than the number of reads with any other type of mismatch for the same position were also excluded.

Strand bias occurs where the mismatch (candidate editing site) occurs preferentially on Read 1 or Read 2 of the aligned read pair. Therefore we excluded positions with Fisher Strand bias, using a Benjamini Hochberg corrected p-value <0.05. Samtools Variant Distance Bias (VDB) and Read Position Bias (RPB) have also been shown to be useful in discriminating true editing sites from false positives. We manually inspected sites with 0.01>VDB>0 and RPB<0.01 and removed 50 additional positions.

**Validation primers for Targeted Deep re-sequencing**

After cDNA synthesis, PCR was performed in a 96-well plate using one primer pair per sample. PCR reactions were set up as follows: 10 μL of Thermo Phusion MM polymerase, 1 μL each of the F and R primers (0.5 μM final concentration), 2 μL of cDNA template and 6 μL of DNAse/RNAse-free water. Cycling conditions were: 120 s at 98°C followed by 30 cycles of 98°C for 10 s, 62°C for 15 s, 72°C 15 s; and a final cycle of 72°C 300 s. PCR products were quantified using a Qubit™ 3.0 Fluorometer (ThermoFisher Scientific Inc., Waltham, MA, USA). 5 μL of each PCR product was electrophoresed in a 3% agarose TBE gel (pre-stained with Biotium Gel-Green) run at 75/100 V for 40 min and visualised under blue light in a Biorad Chemi-Doc Touch imaging system (Biorad, Hercules, CA, USA).

**Supplementary Table 1: mouse samples used for RNA-seq experiments.**

| **Genotype** | **Mouse**  **ID** | **Total**  **raw reads** | **Total**  **trimmed reads** | **Mapped reads Star** | **Mapped reads bowtie1** |
| --- | --- | --- | --- | --- | --- |
| WT | 106 | 61793296 | 60471868 | 45802583 | 27624300 |
| WT | 107 | 42573358 | 41597008 | 31821538 | 19894308 |
| WT | 119 | 50978994 | 50134268 | 35464987 | 26423352 |
| KO | 112 | 54468812 | 53539766 | 41732813 | 25984354 |
| KO | 118 | 47275224 | 46363032 | 42359701 | 21016090 |
| KO | 126 | 41597572 | 40783610 | 32503456 | 19304188 |

**Supplementary Table 2: PCR primers used in targeted deep-resequencing.**

| **Serial Number** | **Oligo**  **Name *** | **Melting Temp** | **Oligo number** | **Sequence** | **Product Size** | **Species** |
| --- | --- | --- | --- | --- | --- | --- |
| E00433 | Ttbk2_ideal_left |  | JM417 | GGTTTCTCTGTGTGGCCCTA |  | Mouse |
| E00434 | Ttbk2_ideal_right |  | JM418 | TGCTGTCCTGATACAGAGATGAA |  | Mouse |
| E00478 | JM462_Tbc1d24_for | 58.8 | JM462 | CATCAGAATTGCTGAGATGGA | 145 | Mouse |
| E00479 | JM463_Tbc1d24_rev | 59.8 | JM463 | GGATGGCCTCAAACTCTCTG | 145 | Mouse |
| E00480 | JM464_Nipa1_for | 60.3 | JM464 | CCAGGTAGAAGGGGCATTTT | 169 | Mouse |
| E00481 | JM465_Nipa_rev | 59.8 | JM465 | TGGGCTGAGACAGGATTTCT | 169 | Mouse |
| E00482 | JM466_Wipi2_for | 60.3 | JM466 | GTAGAGCCAAGCCTCAGCAG | 157 | Mouse |
| E00483 | JM467_Wipi2_rev | 61.26 | JM467 | CCAGGTTTGCCCAGGAGTAT | 157 | Mouse |
| E00484 | JM468_Sh2d5_for | 58.23 | JM468 | TCCACCCAAAAGTACAGACTTC | 195 | Mouse |
| E00485 | JM469_Sh2d5_rev | 58.97 | JM469 | TCTTGTTCTTGTTCTTGTTGTTGA | 195 | Mouse |
| E00486 | JM470_Ezh1_for | 60 | JM470 | TGGTGGGTTTTGCAAGTGTA | 166 | Mouse |
| E00487 | JM471_Ezh1_rev | 59.51 | JM471 | GGATGTCCAAGGACAGAAGG | 166 | Mouse |
| E00488 | JM472_Crk_for | 59.5 | JM472 | TCCAAATGCACAGTCAATCC | 159 | Mouse |
| E00489 | JM473_Crk_rev | 59.87 | JM473 | GGTCTCACTTTCTAACTCCTGACTG | 159 | Mouse |
| E00490 | JM474_Zfp612_for | 58.24 | JM474 | AAATGTATATTTGTTTTTCAAGACAGG | 155 | Mouse |
| E00491 | JM475_zfp612_rev | 59.26 | JM475 | TTAGTTGGGTGGTGGTGATG | 155 | Mouse |
| E00492 | JM476_Pcdh9_for | 59.8 | JM476 | AACATCCCCTTCCTTTTTCAA | 186 | Mouse |
| E00493 | JM477_Pcdh9_rev | 60.91 | JM477 | GGGTGGTGGGGAAGAAAATA | 186 | Mouse |
| E00494 | JM478_Blcap_for | 58.81 | JM478 | AGAGTCGAAGAAAGCGAAGG | 186 | Mouse |
| E00495 | JM479_Blcap_rev | 60.86 | JM479 | GGAGTGGCTGAACCACAGAG | 186 | Mouse |
| E00496 | JM480_Gabra3_for | 58.14 | JM480 | TCTGGCTTAATAGAGAATCTGTCC | 205 | Mouse |
| E00497 | JM481_Gabra3_rev | 60.75 | JM481 | CGCTTGGTGAAGTAGTTGACAGT | 205 | Mouse |
| E00498 | JM482_5HT2CR _for | 59.96 | JM482 | AGATATTTGTGCCCCGTCTG | 178 | Mouse |
| E00506 | JM483_5HT2CR_rev | 63.6 | JM483 | TTGATATTGCCCAAACGATG | 178 | Mouse |

**Table 3: primer sets used for qPCR validation**

| **Primer Name** | **Primer** | **Tm** | **Sequence** | **UPL**  **probe #** | **amplicon**  **length** | **PCR**  **efficiency %** | **Assay**  **type** |
| --- | --- | --- | --- | --- | --- | --- | --- |
| Egr1_left | left | 59 | CCTATGAGCACCTGACCACA | 22 | 90 | 94.21 | UPL |
| Egr1_right | right | 60 | TCGTTTGGCTGGGATAACTC | 22 | 90 | 94.21 | UPL |
| Prrc2a_left | left | 59 | TCTATGGCAGCCCTGGAC | 3 | 76 | 98.23 | UPL |
| Prrc2a_right | right | 60 | TTGGGATGTAACTGAGAGTCGAT | 3 | 76 | 98.23 | UPL |
| Sipa1l3_left | left | 60 | AGAGATCCCTCCTGCACACA | 1 | 112 | 101.26 | UPL |
| Sipa1l3_right | right | 60 | TGTCAGATCCAGGGGTGACT | 1 | 112 | 101.26 | UPL |
| Shank1_left | left | 59 | CCAGTTACATCCCAGAGAGGAC | 91 | 95 | 94.28 | UPL |
| Shank1_right | right | 59 | GATGCTGCTGCTGGGTTT | 91 | 95 | 94.28 | UPL |
| Auts2_left | left | 59 | TCCGTTAGTAAAGATGACAAGGAA | 60 | 113 | 94.17 | UPL |
| Auts2_right | right | 60 | GTGGAGCTGCGGTTATGG | 60 | 113 | 94.17 | UPL |
| Dkkl1_left | left | 59 | TCCAAAGCTTTAGTCGACTGTTC | 52 | 91 | 103.63 | UPL |
| Dkkl1_right | right | 60 | GGAAGGTCTCGGAAGTCCAT | 52 | 91 | 103.63 | UPL |
| Rps16_left | left | 59 | CAAATTTATGCCATCCGACA | 81 | 83 | 99.62 | UPL |
| Rps16_right | right | 59 | TCCTTCTTGGAGGCTTCATC | 81 | 83 | 99.62 | UPL |
| Kcnc3_left | left | 60 | GTGGAGACGGAACCCTTCTT | 97 | 61 | 97.63 | UPL |
| Kcnc3_right | right | 59 | CAAAGGTGAACCAGACCACA | 97 | 61 | 97.63 | UPL |
| Nfix_left | left | 59 | CCTTCCCACGTAACACCACT | 1 | 91 | 98.44 | UPL |
| Nfix_right | right | 59 | CTGGGCAAATCGACCTGT | 1 | 91 | 98.44 | UPL |
| Synpo_left | left | 59 | GAAGAGGCCGATTGACAGAG | 26 | 89 | 90.98 | UPL |
| Synpo_right | right | 60 | TTCGGTGAAGCTTGTGCTC | 26 | 89 | 90.98 | UPL |
| Prrc2b_left | left | 60 | TGCAGGGACACTACGTTCAA | 10 | 119 | 100 | UPL |
| Prrc2b_right | right | 60 | CTTGATTGCTCCGGTTCG | 10 | 119 | 100 | UPL |
| Shank3_left | left | 59 | CTTCAAGACGCGCTCAACTA | 75 | 77 | 101.26 | UPL |
| Shank3_right | right | 59 | CGCTCTTCATCCAGGAACTT | 75 | 77 | 101.26 | UPL |
| Dagla_left | left | 60 | GACAGCCTCTTCAACCTGGA | 12 | 67 | 98 | UPL |
| Dagla_right | right | 60 | CAGGCACCATCATGCAGTAG | 12 | 67 | 98 | UPL |
| IRS2_left | left | 60 | CCACAGTTCAGAGACCTTTTCC | 77 | 84 | 94.55 | UPL |
| IRS2_right | right | 60 | GAATTGTGGCGCTTGGAAT | 77 | 84 | 94.55 | UPL |
| Hprt_left | left | 59 | TCCTCCTCAGACCGCTTTT | 95 | 90 | 95.68 | UPL |
| Hprt_right | right | 59 | CCTGGTTCATCATCGCTAATC | 95 | 90 | 95.68 | UPL |
| Pgbd1_left | left | 60 | ATCCTCAACCACTGCAGGAA | 12 | 74 | 94.92 | UPL |
| Pgbd1_right | right | 59 | TGGAGACTGCCTTTTCAGTG | 12 | 74 | 94.92 | UPL |
| Cbfa2t3_left | left | 59 | GATCACAGGCTCACAGAACG | 29 | 74 | 99.25 | UPL |
| Cbfa2t3_right | right | 60 | TCCATGATGCAGTTCAGAAGAC | 29 | 74 | 99.25 | UPL |

**Supplementary Figures**

**Supplementary Figure 1**

**
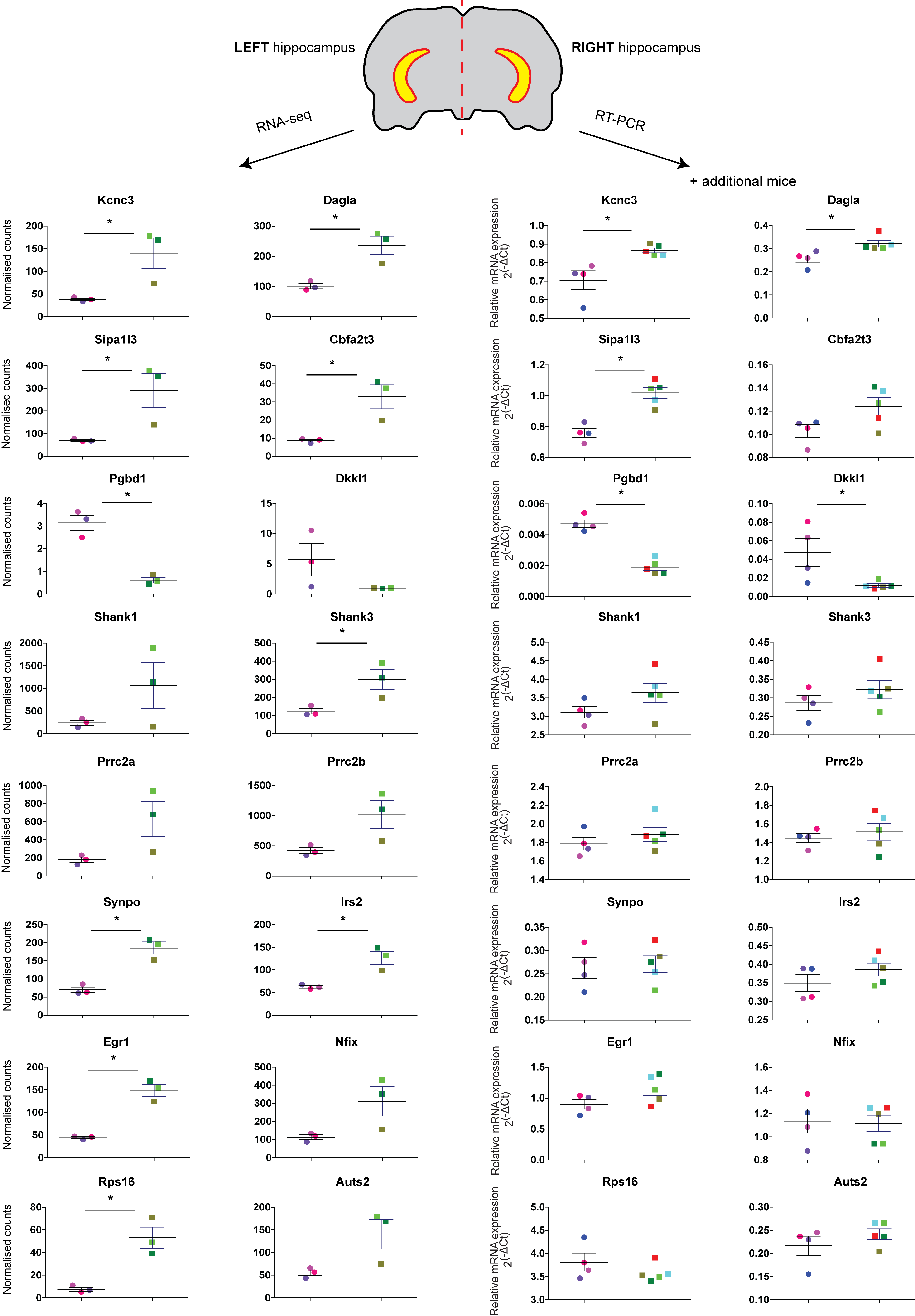
**

**Supplementary Figure 1**: **Subtle modulation of hippocampal gene expression in *Adar3^exon3^* mice.** Normalised counts (RNAseq experiment) (n=3/genotype) and relative mRNA expression (RT-PCR) (n= 4 WT, n=5 *Adar3^exon3^*) of 16 genes from the left or right hippocampus respectively. Gene expression (RT-PCR) was normalized to *Hprt*. Error bars indicate SEM. Star (*) indicates P<0.05.

**Supplementary Figure 2**


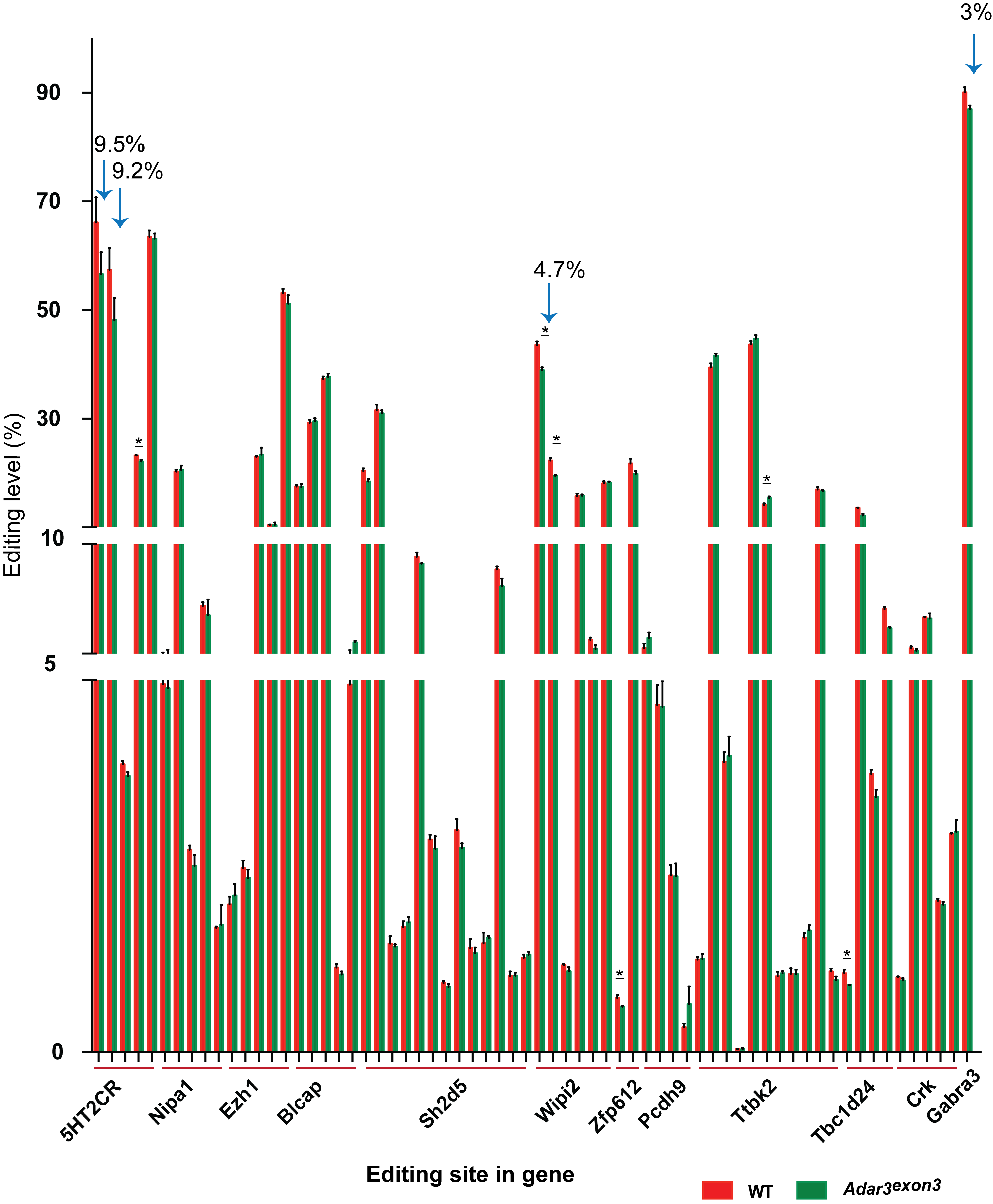


**Supplementary Figure 2: *Adar3* deficiency does not change substantially the editing frequencies of 66 editing sites in 12 deeply sequenced genetic regions.** RNA editing levels of editing sites in the 12 targeted regions for re-sequencing (66 sites, see methods and Supplementary File 1) (n=3/genotype). The largest differences in editing levels are marked with blue arrows. Error bars indicate SEM. Star (*) indicates P<0.05.

**Supplementary Figure 3**


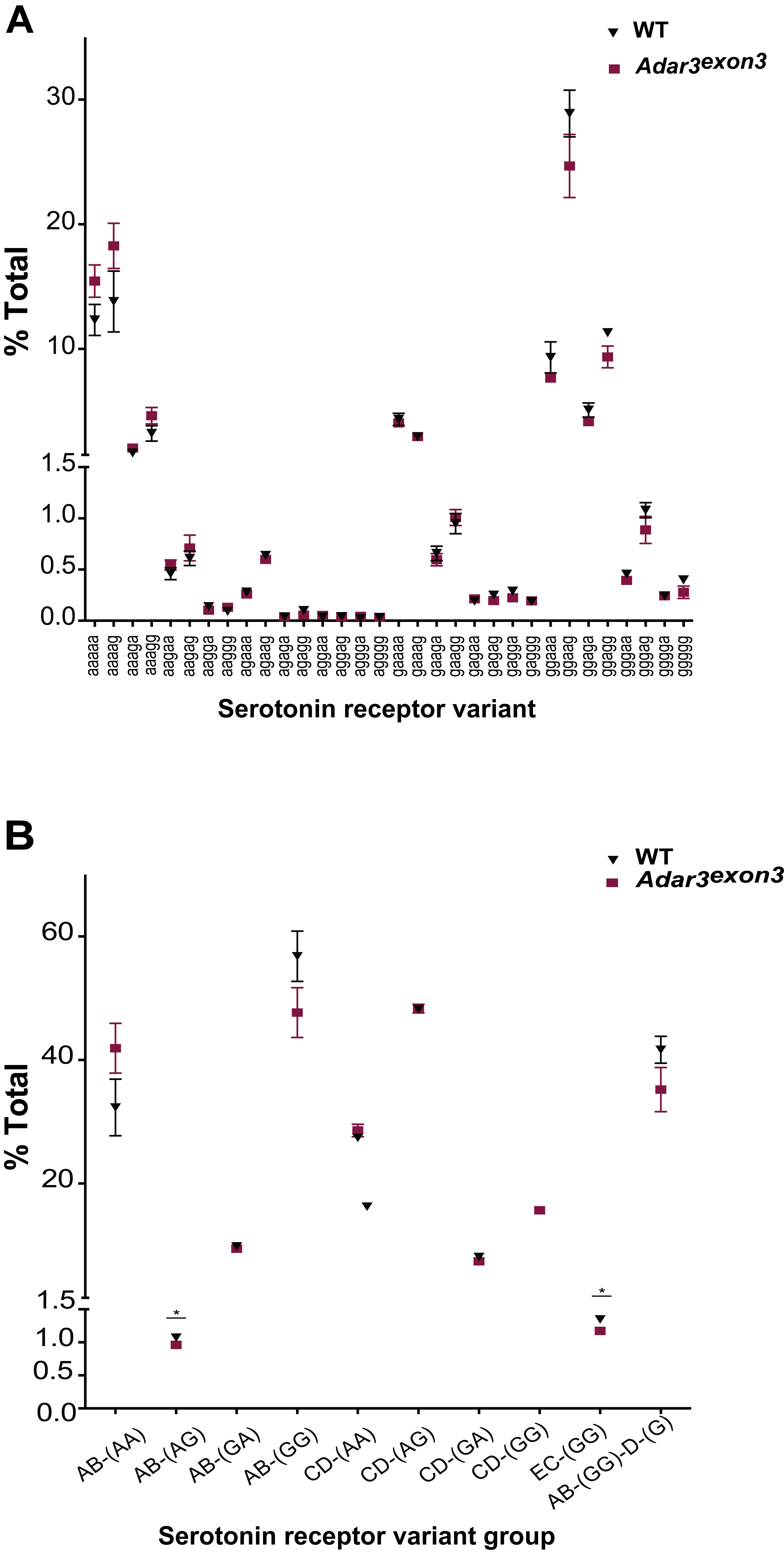


**Supplementary Figure 3: *Adar3* deficiency does not modulate the frequency of 32 5-HT2CR serotonin receptor mRNA variants, resulting from editing on 5 sites. (A)** Comparison of transcript variant frequencies in the hippocampus between WT and *Adar3^exon3^* animals (see also Supplementary File 2) **(B)** Comparison of transcript variant *group* frequencies in the hippocampus between WT and *Adar3^exon3^* animals. Groups: AB-(AA) - fully unedited A and B sites, AB-(AG) - A site unedited, B site edited, AB-(GA) - A site edited, B site unedited, AB-(GG) – fully edited A and B sites, CD-(AA) – fully unedited C and D sites, CD-(GA) – C site edited, D site unedited, CD-(AG) – C site unedited, D site edited, CD-(GG) – fully edited C and D sites, EC-(GG) - fully edited E and C sites, AB-(GG)-D-(G) – fully edited A and B and D sites. (n=3/genotype) Error bars indicate SEM. Star (*) indicates P<0.05.

**Supplementary Figure 4**


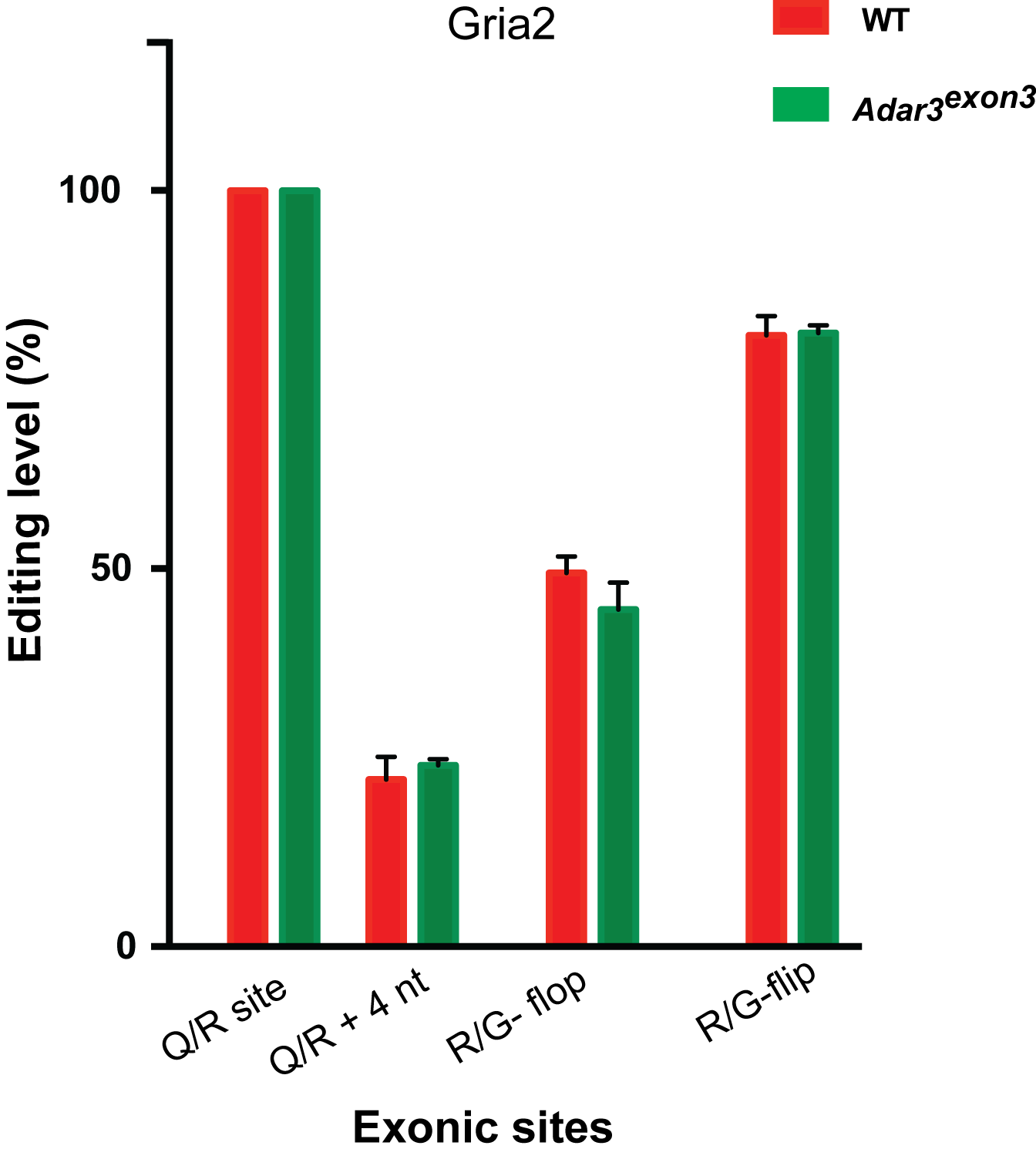


**Supplementary Figure 4: *Adar3* deficiency has no effect on the editing levels of the *Gria2* Q/R or R/G sites in the mouse hippocampus.** RNA editing analysis for the editing frequencies of the exonic Q/R site, Q/R site + 4 nt, and the R/G site in the flip or the flop isoform of *Gria2* from the RNA-seq experiment. Error bars indicate SEM.

**References**

Langmead, B., Trapnell, C., Pop, M., and Salzberg, S.L. (2009). Ultrafast and memory-efficient alignment of short DNA sequences to the human genome. *Genome Biol* 10(3)**,** R25. doi: 10.1186/gb-2009-10-3-r25.

Li, H., Handsaker, B., Wysoker, A., Fennell, T., Ruan, J., Homer, N., et al. (2009). The sequence alignment/map format and SAMtools. *Bioinformatics* 25(16)**,** 2078-2079. doi: 10.1093/bioinformatics/btp352.
